# Author Correction: A systems biology approach uncovers cell-specific gene regulatory effects of genetic associations in multiple sclerosis

**DOI:** 10.1038/s41467-019-10951-1

**Published:** 2019-07-01

**Authors:** International Multiple Sclerosis Genetics Consortium, Lohith Madireddy, Nikolaos A. Patsopoulos, Chris Cotsapas, Steffan D. Bos, Ashley Beecham, Jacob McCauley, Kicheol Kim, Xiaoming Jia, Adam Santaniello, Stacy J. Caillier, Till F. M. Andlauer, Lisa F. Barcellos, Tone Berge, Luisa Bernardinelli, Filippo Martinelli-Boneschi, David R. Booth, Farren Briggs, Elisabeth G. Celius, Manuel Comabella, Giancarlo Comi, Bruce A. C. Cree, Sandra D’Alfonso, Katrina Dedham, Pierre Duquette, Efthimios Dardiotis, Federica Esposito, Bertrand Fontaine, Christiane Gasperi, An Goris, Bénédicte Dubois, Pierre-Antoine Gourraud, Georgios Hadjigeorgiou, Jonathan Haines, Clive Hawkins, Bernhard Hemmer, Rogier Hintzen, Dana Horakova, Noriko Isobe, Seema Kalra, Jun-ichi Kira, Michael Khalil, Ingrid Kockum, Christina M. Lill, Matthew R. Lincoln, Felix Luessi, Roland Martin, Annette Oturai, Aarno Palotie, Margaret A. Pericak-Vance, Roland Henry, Janna Saarela, Adrian Ivinson, Tomas Olsson, Bruce V. Taylor, Graeme J. Stewart, Hanne F. Harbo, Alastair Compston, Stephen L. Hauser, David A. Hafler, Frauke Zipp, Philip De Jager, Stephen Sawcer, Jorge R. Oksenberg, Sergio E. Baranzini

**Affiliations:** 1 Weill Institute for Neurosciences, Department of Neurology, University of California San Francisco, San Francisco, CA 94158 USA; 2 Systems Biology and Computer Science Program, Ann Romney Center for Neurological Diseases, Department of Neurology, and Division of Genetics, Department of Medicine, Brigham & Women’s Hospital, Harvard Medical School, Boston, MA 02115 USA; 3 Broad Institute of Harvard University and Massachusetts Institute of Technology, Cambridge, MA 02142 USA; 4 Departments of Neurology, Yale School of Medicine, 300 George St, New Haven, CT 06511 USA; 5 Department of Genetics, Yale School of Medicine, 300 George St, New Haven, CT 06511 USA; 6 Institute of Clinical Medicine, University of Oslo, Oslo, 0318 Norway; 7 Department of Neurology, Oslo University Hospital, Oslo, 0424 Norway; 8 John P. Hussman Institute for Human Genomics, University of Miami, Miller School of Medicine, Miami, FL 33136 USA; 9 Dr. John T. Macdonald Foundation Department of Human Genetics, University of Miami, Miller School of Medicine, Miami, FL 33136 USA; 10 Department of Neurology, Klinikum rechts der Isar, School of Medicine, Technical University of Munich, 81675 Munich, Germany; 11 Munich Cluster for Systems Neurology (SyNergy), 81377 Munich, Germany; 12 Division of Epidemiology, School of Public HealthUniversity of California, 324 Stanley Hall, MC#3220, Berkeley, CA 94720 USA; 13 Department of Mechanical, Electronics and Chemical Engineering, Oslo Metropolitan University, Oslo, 0167 Norway; 14 Section of Biostatistics, Neurophyisiology and Psychiatry, Unit of medical and genomic statistics, Universita of Pavia, Pavia, 27100 Italy; 15 Department of Biomedical Sciences for Health, University of Milan, Milan, 20133 Italy; 16 MS Research Unit and Department of Neurology, IRCCS Policlinico San Donato, San Donato Milanese, Milan, 20097 Italy; 17 Faculty of Medicine, Westmead Clinical School, The Westmead Institute for Medical Research, Sydney, NSW 2145 Australia; 18 Department of Quantitative and Population Health Sciences, School of Medicine, Case Western Reserve University, Cleveland, OH 44106 USA; 19 Institute of Health and Society, University of Oslo, Oslo, 0318 Norway; 20 Servei de Neurologia-Neuroimmunologia. Centre d’Esclerosi Múltiple de Catalunya (Cemcat), Institut de Recerca Vall d’Hebron (VHIR), Hospital Universitari Vall d’Hebron, Universitat Autònoma de Barcelona, Barcelona, 08035 Spain; 21 Department of Neurology, San Raffaele Scientific Institute, Milan, 20132 Italy; 22 Department of Health Sciences, UPO University, Novara, 28100 Italy; 23 Department of Clinical Neurosciences. Neurology Unit, University of Cambridge, Cambridge, CB2 1QW UK; 24 Faculté de médecine, MS Clinic Centre Hospitalier de l’, Université de Montréal. Université de Montréal Montreal, Montreal, QC H3A 1G1 Canada; 25 Department of Neurology, Laboratory of Neurogenetics, University of Thessaly, University Hospital of Larissa, Larissa, 41223 Greece; 26 Department of Neurology, University Hopital Pitié-Salpêtrière, Paris, 75013 France; 27 UMR 1127, Sorbonne-Université, INSERM, University Hopital Pitié-Salpêtrière, Paris, 75013 France; 28 KU Leuven Department of Neurosciences, Laboratory for Neuroimmunology, Leuven, 3000 Belgium; 29 Université de Nantes, INSERM, Centre de Recherche en Transplantation et Immunologie, UMR 1064, ATIP-Avenir, Equipe 5, Nantes, F-44093 France; 30 CHU de Nantes, INSERM, CIC 1413, Pôle Hospitalo-Universitaire 11: Santé Publique, Clinique des données, Nantes, F-44093 France; 31 Department of Neurology, Medical School, University of Cyprus, Nicosia, 587G+X2 Cyprus; 32 Institute for Science & Technology in Medicine, Keele University, Keele, ST5 5GB UK; 33 Department of Neurology, Erasmus MC Dr Molewaterplein 40, Rotterdam, 3015 GD The Netherlands; 34 Department of Immunology, Erasmus MC, Rotterdam, 3015 GD The Netherlands; 35 First Faculty of Medicine, Department of Neurology and Center of Clinical Neuroscience, Charles University and General University Hospital, Prague, 3CFG+RJ Czech Republic; 36 Department of Neurology, Kyushu University, Kyushu, 812-0053 Japan; 37 Royal Stoke MS Centre of Excellence, University Hospital North Midlands, Stoke-on-Trent, ST4 6QG UK; 38 Department of Neurology, Medical University of Graz, Graz, A-8036 Austria; 39 Department of Clinical Neuroscience and Center for Molecular Medicine, Karolinska Institutet, Stockholm, 17176 Sweden; 40 Department of Neurology, University Medical Center of the Johannes Gutenberg University Mainz, Mainz, 55131 Germany; 41 Genetic and Molecular Epidemiology Group, Lübeck Interdisciplinary Platform for Genome Analytics, Institutes of Neurogenetics and Cardiogenetics, University of Lübeck, Lübeck, 23562 Germany; 42 Neuroimmunology and MS Research (nims), Department of Neurology, University Zurich, Zürich, 8006 Switzerland; 43 Department of Neurology, section 2082, Rigshospitalet, Danish Multiple Sclerosis Center, University of Copenhagen, Copenhagen, 2100 Denmark; 44 Institute for Molecular Medicine Finland (FIMM), University of Helsinki, Helsinki, FIN-00014 Finland; 45 Harvard University, Center for Human Genetic Research, Boston, MA 02115 USA; 46 UK Dementia Research Institute at University College London, London, WC1E6BT UK; 47 Menzies Institute for Medical Research, University of Tasmania, Hobart, TAS 7000 Australia; 48 Department of Clinical Neurosciences, Cambridge Biomedical Campus, University of Cambridge, Cambridge, CB2 0QQ UK; 49 Department of Neurology, Center for Translational and Computational Neuroimmunology and Multiple Sclerosis Center, Columbia University Medical Center, New York, NY 10032 USA; 50 Bakar Institute for Computational Health Science. University of California San Francisco, San Francisco, CA 94158 USA

**Keywords:** Cellular signalling networks, Genome informatics, Genome-wide association studies, Multiple sclerosis

Correction to: *Nature Communications* 10.1038/s41467-019-09773-y, published online 20 May 2019.

The original version of this Article contained an error in the spelling of the author Nikolaos A. Patsopoulos, which was incorrectly given as Niklaos A. Patsopoulos, and author Efthimios Dardiotis, which was incorrectly given as Dardiotis Efthimios. This has now been corrected in both the PDF and HTML versions of the Article.

