# Supplementary material for: A systems biology approach uncovers cell-specific gene regulatory effects of genetic associations in multiple sclerosis
Source: Nat Commun. 2019 May 20;10:2236. doi: 10.1038/s41467-019-09773-y (PMC6527683; doi:10.1038/s41467-019-09773-y)
Supplement: Supplementary file 1 — Supplementary information [file 41467_2019_9773_MOESM1_ESM.pdf]

Supplementary Information

A Systems Biology approach to determine cell-specific gene regulatory effects of genetic associations in multiple sclerosis

International Multiple Sclerosis Genetics Consortium*

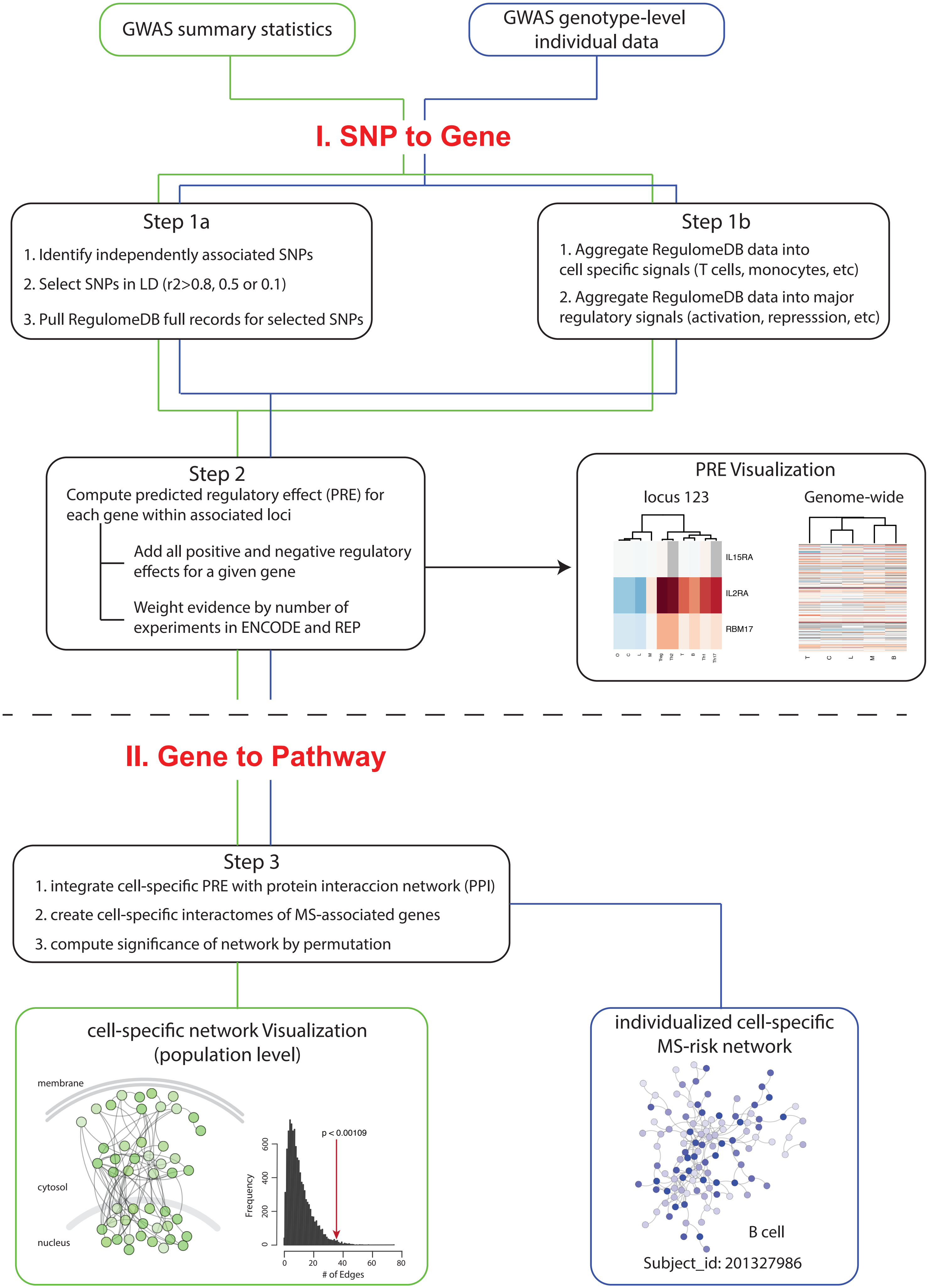

Supplementary Figure 1

Supplementary Figure 1. Overview of our approach. Both summary statistics and genotype-level data were incorporated into the pipeline. The algorithm consists of two stages: I. SNP to Gene (steps 1 and 2) and II. Gene to pathway (step 3). Step 1 consists in selecting individual SNPs, and those in LD. Subsequently, RegulomeDB records for all selected SNPs are parsed and organized in discrete regulatory signals (activation, repression, etc) and discrete cell type (B cell, T cell, etc) where the regulatory effect has been documented. Step 2 consists in computing an aggregate score for each nearby gene from all regulatory signals. This predicted regulatory effect (PRE) is then used to prioritize each gene within a region and construct a local protein interactome based on an established protein interaction network. Finally, the resulting network is evaluated for statistical significance and visualized both at the population and individual level.

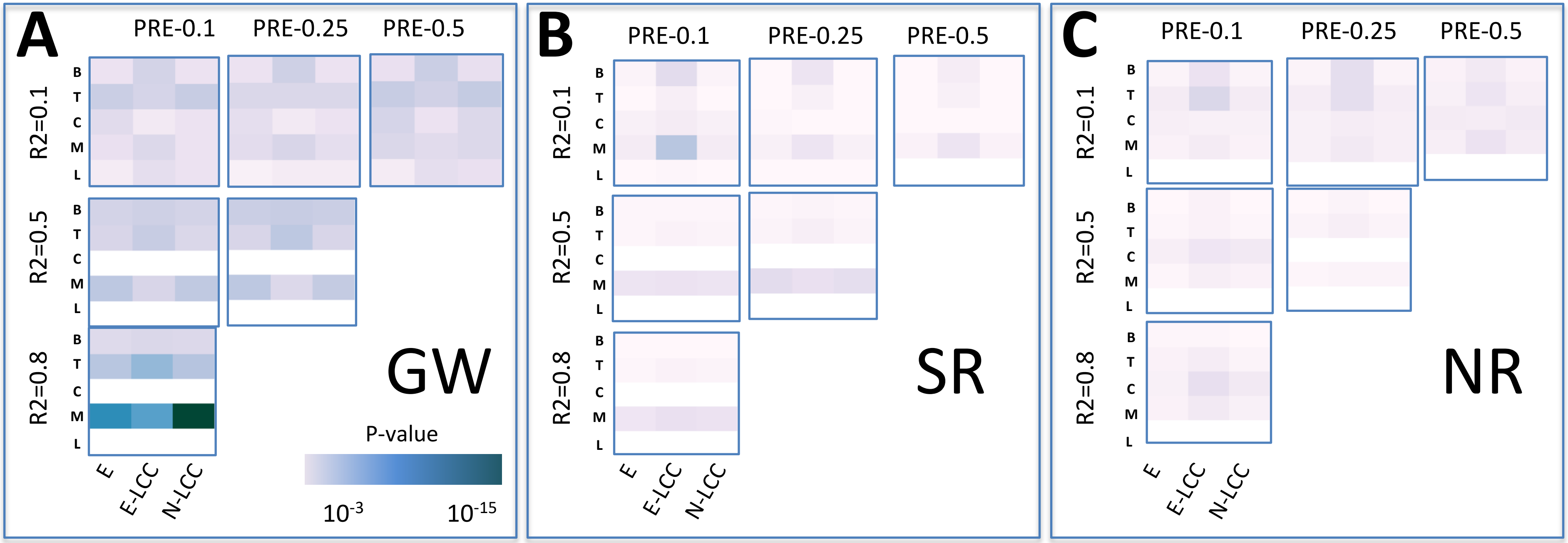

Supplementary Figure 2

Supplementary Figure 2. Sensitivity analysis of cell-specific sub-networks. Basic topological measures such as the number of edges of the entire sub-network (E) and the number of nodes (N-ECC) and edges (E-LCC) in the largest connected component were computed for networks generated using different PRE thresholds (10th, 25th, and 50th percentiles), and association r^2 cut-offs (0.1, 0.5 and 0.8) for each cell type (B: B cells; T: T cells; C: CNS; M: monocytes; L: lung) at each level of confidence (GW, SR and NR). Each colored cell represents the significance of each tested network metric. Panel A shows that significant networks were obtained across a wide spectrum of conditions (the lower value observed at the 99th percentile is due to the small networks resulting from using that extreme PRE threshold). Panels B and C show that the significance of generated networks with SR and NR variants is much lower than with GW variants.

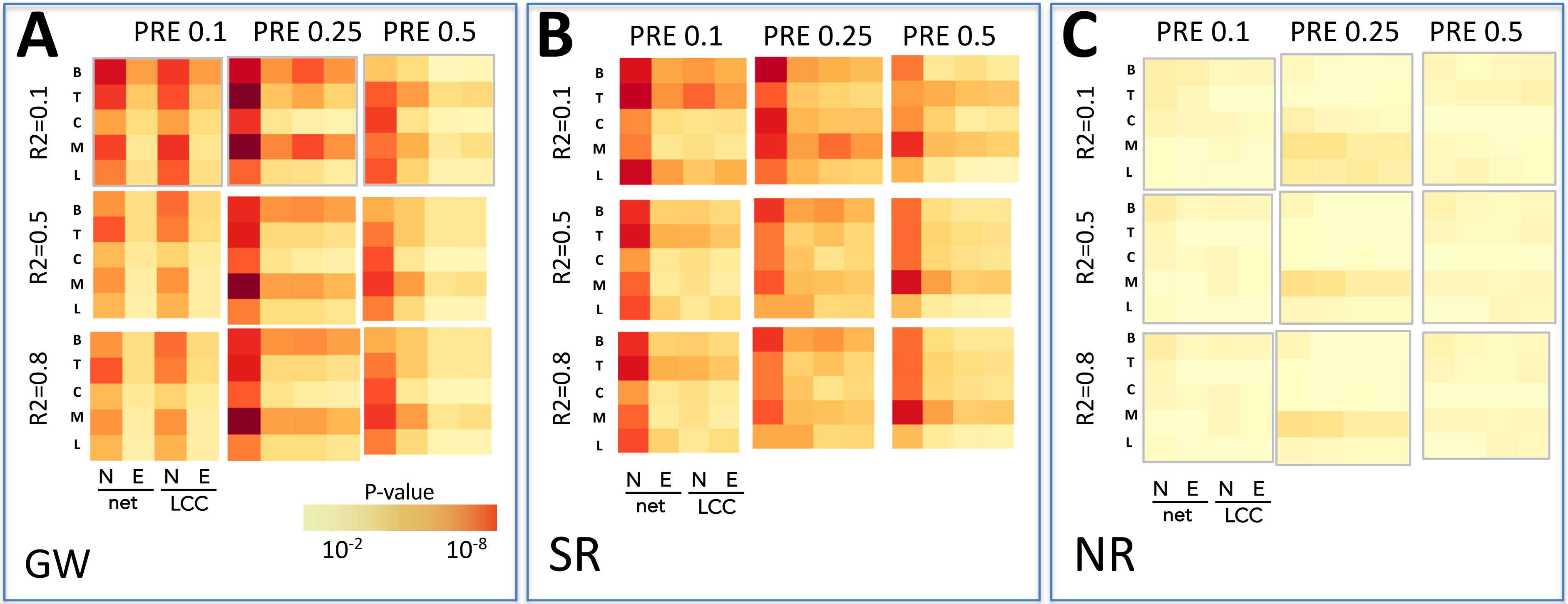

Supplementary Figure 3

Supplementary Figure 3. Sensitivity analysis of individual-level networks. Basic topological measures such as the number of nodes (N) and edges (E) of the entire sub-network and the number of nodes (N-ECC) and edges (E-LCC) in the largest connected component were computed for networks generated using different PRE thresholds (10th, 25th, and 50th percentiles), and association r^2 cut-offs (0.1, 0.5 and 0.8) for each cell type (B: B cells; T: T cells; C: CNS; M: monocytes; L: lung) at each level of confidence (GW, SR and NR). Then, each metric was compared between cases and controls by means of T-test (1-tailed) to test the hypothesis that on average more significant networks are obtained for patients than for healthy controls. Each colored cell represents the significance of each tested network metric. Panel A shows that significant networks were obtained across a wide spectrum of conditions (the lower value observed at the 50th percentile is due to that only small networks result from using that extreme PRE threshold). Panel B shows that using variants from SR regions yields networks with significant differences between cases and controls. Panel C show that NR regions do not result in significant differences between cases and controls.

Cell Type	Corr (r)	pval
B	0.286	$8.18\ 10^{-54}$
T	0.338	$9.27\ 10^{-76}$
C	0.158	$2.10\ 10^{-17}$
L	0.162	$3.73\ 10^{-18}$
M	0.358	$5.07\ 10^{-85}$

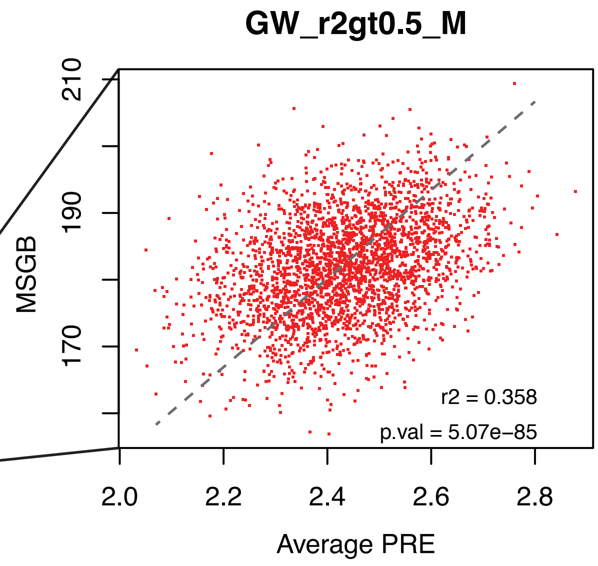

Supplementary Figure 4

Supplementary Figure 4: Individual PREs correlate with global polygenic risk scores: Cell-specific predicted regulatory effects (PRE) of 2,370 MS cases were correlated with their corresponding polygenic risk scores (MS genetic burden, MSGB). The table shows all correlations between MSGC and PRE for each cell type discussed in this paper. A representative scatter plot shows individual values of MSGB (Y-axis) and average PRE for monocytes (X-axis).

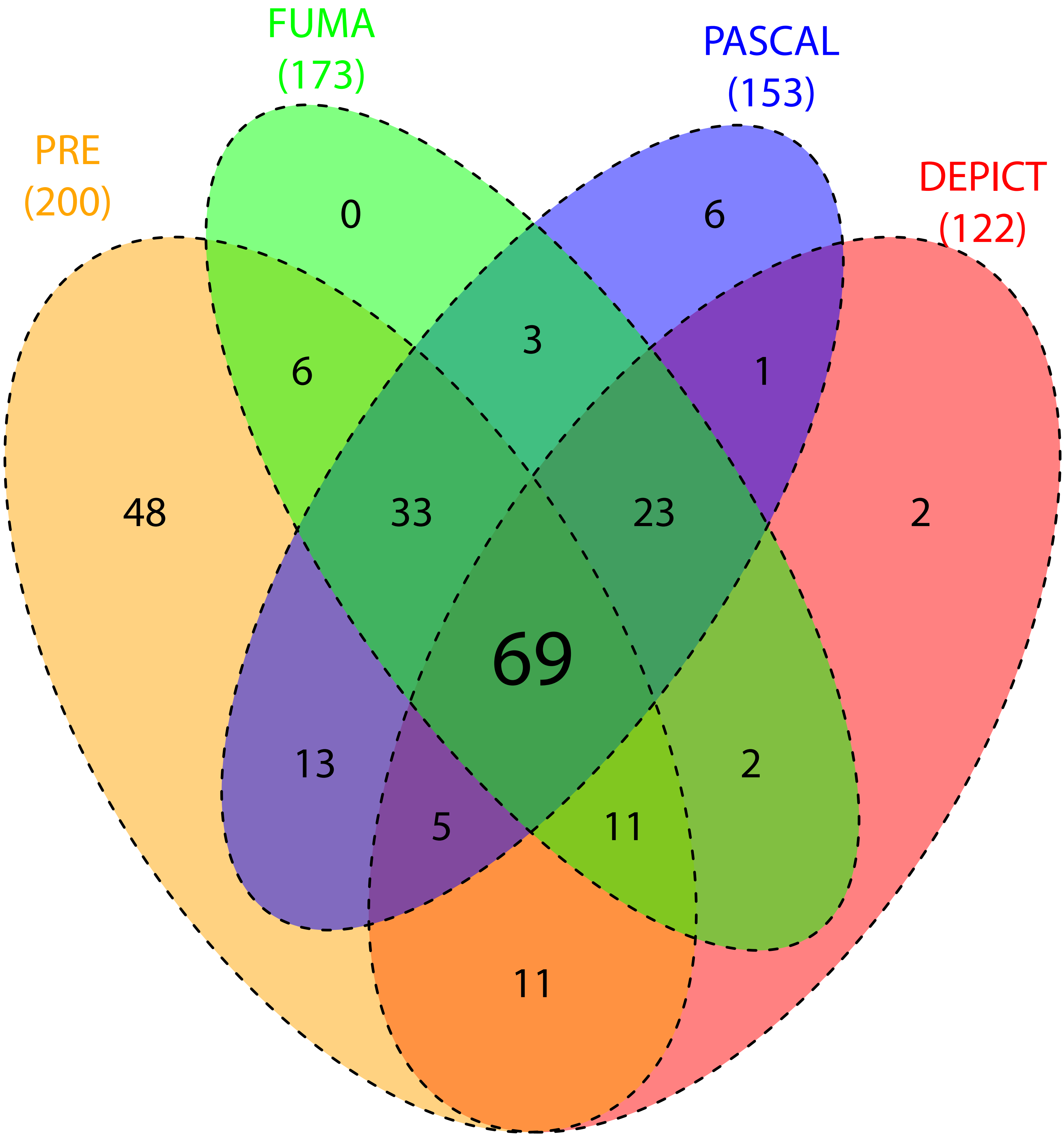

Supplementary Figure 5

Supplementary Figure 5: Comparison of gene prioritization across three methods: SNP to gene operations were computed in FUMA, DEPICT, and PASCAL and the results were compared to those obtained in this work. For 69 out of 200 loci the prioritized genes were the same across all methods. The method presented here produced a gene output for every association and shared the most genes with the other three methods (119 with FUMA, 96 with DEPICT, and 120 with PASCAL).

Supplementary Table 1. Feature comparison with other pathway methods

Method	PRE	DEPICT	FUMA	PASCAL
SNP to gene mapping per cell/tissue type	Yes	No	Yes	No
Cell type/Tissue specific gene prioritization	Yes	No	Yes	No
Leverages SNP Regulatory information	Yes	No	Yes	No
Takes into account SNPs in LD	Yes	Yes	Yes	Yes
Requires SNP p-values as input	No	No	Yes	Yes
SNP filtering based on predicted functional annotations from tools	No	No	Yes	No
Genes assignment by proximity	No	Yes	yes	Yes
Needs predefined gene sets	Yes	Yes	Yes	Yes
computes individual regulatory burden	Yes	No	No	No
Enrichment analysis of prioritized genes in biological pathways	Yes	Yes	Yes	Yes
Default restriction based on MAF	No	Yes	Yes	Yes

Supplementary Table 2. Comparison of results with other pathway methods

Effect	PRE	Depict	Fuma
chr11:118783424	DDX6	NA	NA
chr11:14868316	RRAS2	NA	NA
chr13:100026952	MIR548AN|TM9SF2	NA	NA
chr14:88523488	GALC	NA	NA
chr16:11213951	CLEC16A	NA	NA
chr16:11353879	CLEC16A	NA	NA
chr1:154983036	FLAD1	NA	NA
chr1:32738415	FAM167B|LCK	NA	NA
chr2:112492986	ANAPC1|MERTK	NA	NA
chr3:100848597	LOC152225|SENP7|ZPLD1	NA	NA
chr3:112693983	CD200R1	NA	NA
chr3:121765368	IQCB1	NA	NA
chr3:121783015	IQCB1	NA	NA
chr5:40429250	DAB2|PTGER4	NA	NA
chr6:119215402	FAM184A|MCM9	NA	NA
chr6:130348257	L3MBTL3	NA	NA
chr6:14691215	CD83|JARID2|NA	NA	NA
chr7:50328339	C7orf72|IKZF1	NA	NA
chr8:129177769	LINC00977|MIR1208|PCAT1|POU5F1B	NA	NA
chr8:95851818	INTS8	NA	NA
rs10063294	CAPSL|IL7R	IL7R	IL7R
rs1014486	IL12A|LINC01100	IL12A	IL12A
rs10191360	CXCR4|DARS|THSD7B	CXCR4	CXCR4
rs10230723	C7orf72|IKZF1	NA	NA
rs10245867	JAZF1|JAZF1-AS1	JAZF1	JAZF1
rs1026916	STAT3	STAT3	STAT5A|STAT3|PTRF
rs10271373	ZC3HAV1|ZC3HAV1L	ZC3HAV1	ZC3HAV1L|ZC3HAV1
rs1076928	STK38	ETV7|KCTD20	ETV7|PXT1|KCTD20
rs1077667	C3|TNFSF14	TNFSF14	TNFSF14|C3
rs10801908	CD58	CD58	CD58
rs1087056	ZEB1-AS1|ZNF438	ZNF438	NA
rs10936182	IL12A|LINC01100	IL12A	NA
rs10936602	LRRC31|MYNN	NA	ACTRT3|MYNN|LRRC34|LRRIQ4|LRRC31
rs10951042	CARD11|CHST12|SDK1	CARD11	NA
rs10951154	HOXA1|NA	NA	HOXA1|HOXA2
rs11079784	NPEPPS	NPEPPS	ITGB3|ITGB3|EFCAB13|NPEPPS|KPNB1|TBKBP1
rs11083862	NA	NA	SAE1
rs11125803	CENPO	NCOA1	PTRHD1|CENPO|ADCY3
rs1112718	HHEX|IDE	HHEX	IDE|KIF11|HHEX
rs11161550	BCL10|C1orf52|LOC646626	BCL10	NA
rs11231749	PLCB3|VEGFB	CCDC88B|RPS6KA4|GPR137	NUDT22|DNAJC4|VEGFB|FKBP2|PPP1R14B|PLCB3|BAD|GPR137|KCNK4|TEX40|ESRRA|TRMT112|PRDX5|CCDC88B|RPS6KA4
rs11256593	IL15RA|IL2RA	IL2RA	RBM17
rs11578655	DPH6|SLC30A7	NA	EXTL2|SLC30A7|DPH5|NA
rs11749040	DAB2|PTGER4	PTGER4	NA
rs1177228	KIAA1841|PUS10	PUS10	PUS10|PEX13|KIAA1841
rs11809700	EVI5|FAM69A	NA	RPL5|FAM69A|GFI1|EVI5
rs11852059	FRMD6|GNG2	GNG2	GNG2
rs11899404	MIR4262|NA|TRIB2	NA	NA
rs11919880	CCR4|GLB1	CCR4	TRIM71|CCR4
rs12133753	EVI5|FAM69A	TGFBR3	TGFBR3
rs12147246	TRAF3	TRAF3	TRAF3
rs12211604	RREB1	NA	RREB1
rs12365699	DDX6	CXCR5	DDX6|CXCR5
rs12434551	ZFP36L1	ZFP36L1	ZFP36L1
rs12478539	HAAO|ZFP36L2	NA	NA
rs1250551	ZMIZ1	ZMIZ1	ZMIZ1
rs12588969	TRAF3	TRAF3	TRAF3
rs12609500	SMARCA4	NA	SMARCA4
rs12614091	CD28|CTLA4|RAPH1	CD28	CD28
rs12622670	FBXO48|PLEK	PLEK	PLEK
rs12722559	IL15RA|IL2RA	NA	IL2RA|RBM17
rs12832171	TNFRSF1A|U47924.1	NA	NA
rs12925972	DYNLRB2|WWOX	NA	WWOX
rs12971909	HDGFRP2|NA|UBXN6	NA	UBXN6|HDGFL2
rs13066789	LPP	NA	LPP
rs13136820	CHRNA9|RHOH	NA	NA
rs1323292	RGS1	RGS1	RGS1
rs13327021	CMC1|EOMES	EOMES	EOMES
rs13385171	MIR4778|SPRED2	NA	SPRED2
rs13414105	LBH	NA	NA
rs1365120	PRR5L	PRR5L	PRR5L
rs137955	ENTHD1|GRAP2	ENTHD1|GRAP2	ENTHD1|GRAP2
rs1399180	GATA3|GATA3-AS1|TAF3	GATA3	GATA3
rs140522	ODF3B|SCO2	SCO2	NCAPH2|SCO2|TYMP|ODF3B
rs1415069	EVI5|FAM69A	EVI5|FAM69A	RPL5|FAM69A
rs1465697	SLC6A16	CD37|DKKL1	SLC6A16|CD37|TEAD2|DKKL1|CCDC155|PTH2|GFY
rs17051321	NDNF|TNIP3	TNIP3	TNIP3
rs1738074	RSPH3|TAGAP	TAGAP	RSPH3|TAGAP
rs17724508	DYNLRB2|WWOX	NA	NA
rs17741873	CAMK2G	PLAU	CAMK2G|PLAU|C10orf55
rs17780048	IL20RA|IL22RA2	TNFAIP3	TNFAIP3
rs1800693	TNFRSF1A|U47924.1	NA	PLEKHG6|TNFRSF1A|SCNN1A
rs1801133	CLCN6	NA	C1orf167|MTHFR
rs2084007	JADE2|TCF7	JADE2	JADE2
rs2150879	VMP1	VMP1	VMP1|TUBD1|RPS6KB1|NA|RNFT1
rs2248137	BCAS1	NA	CYP24A1
rs2269434	MADD	SPI1|DDB2|NR1H3|ACP2	DDB2|ACP2|NR1H3|MADD|MYBPC3|SPI1|RAPSN
rs2286974	CLEC16A	NA	CIITA|DEXI|CLEC16A
rs2289746	CBLB	CBLB	CBLB
rs2317231	CD5L|FCRL1|FCRL3	FCRL1|FCRL3	FCRL3|FCRL2|FCRL1
rs2327586	AHI1|MYB	MYB	MYB
rs2331964	IQCB1	IQCB1	FBXO40|HCLS1|IQCB1|EAF2
rs2364485	TNFRSF1A|U47924.1	NA	LTBR
rs244656	JADE2|TCF7	TCF7	TCF7
rs2469434	CD226|NA	CD226	DOK6|CD226
rs249677	NDFIP1	NDFIP1	NDFIP1
rs2546890	LOC285626	IL12B	IL12B
rs2585447	BCAS1	NA	CYP24A1
rs2590438	LPP	BCL6	NA
rs2705616	AFF1|LOC100506746	AFF1	C4orf36|AFF1
rs2726479	PPA2	TET2	TET2|PPA2
rs2836438	ERG	TMPRSS3|ERG	ERG
rs28703878	PKIA	PKIA	NA
rs28834106	SMARCA4	NA	KEAP1
rs2986736	TNFRSF25	TNFRSF25	ESPN|TNFRSF25|PLEKHG5
rs3184504	HECTD4	SH2B3|TRAFD1	SH2B3|ATXN2|BRAP|ACAD10|NA|ALDH2|MAPKAPK5|NAA25|TRAFD1|HECTD4|RPL6|PTPN11
rs32658	TNFAIP8	TNFAIP8	TNFAIP8
rs34026809	DDX6	CXCR5	ARCN1|PHLDB1|TREH
rs34536443	SMARCA4	NA	ZGLP1|FDX1L|NA|NA|RAVER1|TYK2|CDC37|PDE4A
rs34681760	PAPD7	NA	PAPD7
rs34695601	BATF|FLVCR2	BATF	BATF
rs34723276	DPH5|SLC30A7	NA	NA
rs34947566	CLEC16A	RMI2|CLEC16A	RMI2|TNP2|PRM3|PRM2|PRM1|LOC400499
rs35218683	IFITM3	IFITM3	NA
rs354033	ZNF746	ZNF746	ZNF746
rs35486093	BCL10|C1orf52|LOC646626	BCL10	C1orf52|BCL10
rs35540610	SP110|SP140	SP140|SP110	SP110|SP140
rs35703946	IRF8|LINC01082	IRF8	NA
rs3737798	CD48|VANGL2	NA	VANGL2
rs3809627	GDPD3	TBX6|PPP4C|YPEL3	ALDOA|PPP4C|TBX6|YPEL3|GDPD3|MAPK3
rs3923387	PLEC	PARP10|GRINA|PLEC	PLEC|PARP10|GRINA|SPATC1
rs405343	SOX8|SSTR5-AS1	NA	NA
rs4262739	ETS1	ETS1	ETS1
rs4325907	LOC152225|SENP7|ZPLD1	ZPLD1	NA
rs438613	CMC1|EOMES	NA	NA
rs4409785	FAM76B|SESN3	NA	NA
rs4728142	IRF5|KCP	IRF5	IRF5
rs4796224	GGNBP2	NA	ZNHIT3|MYO19|PIGW|GGNBP2|DHRS11|MRM1
rs4808760	MAST3	IFI30|MAST3	IL12RB1|MAST3|PIK3R2|PIK3R2|IFI30|MPV17L2|RAB3A|PDE4C
rs4812772	TOX2	TOX2	TOX2
rs4820955	LIMK2	NA	NA
rs483180	PHGDH	NA	PHGDH
rs4896153	AHI1|MYB	NA	AHI1
rs4939490	CD5|CD6	CD6	CD6
rs4940730	MALT1	ALPK2	ALPK2
rs531612	SART1	CTSW|C11orf68	FIBP|CCDC85B|FOSL1|C11orf68|DRAP1|SART1|EIF1AD|BANF1|CST6|CATSPER1
rs55858457	CARD11|CHST12|SDK1	NA	CHST12
rs56095240	FAM76B|SESN3	NA	NA
rs57116599	ANAPC1|MERTK	MERTK	MERTK|TMEM87B
rs5756405	CSF2RB|NCF4	CSF2RB	CSF2RB|NCF4
rs58166386	EPS15L1	NA	EPS15L1|CALR3|NA|C19orf44
rs58394161	EVI5|FAM69A	NA	C1orf146|GLMN|GFI1|EVI5
rs59655222	C1orf106|KIF21B	KIF21B	C1orf106|KIF21B|CACNA1S
rs6020055	PTPN1	SLC9A8	SLC9A8
rs6032662	CD40|NCOA5|SLC12A5	CD40|NCOA5	SLC12A5|NCOA5|CD40
rs60600003	ELMO1	ELMO1	ELMO1
rs6072343	NA	NA	TOP1|PLCG1|ZHX3|LPIN3|EMILIN3
rs61708525	PLXNC1	PLXNC1	PLXNC1
rs61863928	ADO|ZNF365	ADO	NA
rs61884005	RRAS2	RRAS2|PDE3B	RRAS2|COPB1|PSMA1|PSMA1
rs62013236	CTSH	CTSH	CTSH|RASGRF1
rs62420820	IL20RA|IL22RA2	IL22RA2	NA
rs631204	IL20RA|IL22RA2	NA	NA
rs6427540	CD48|VANGL2	SLAMF1	SLAMF1|CD48
rs6496663	IQGAP1	IQGAP1	GABARAPL3|ZNF774|IQGAP1
rs6533052	UBE2D3	SLC9B2|UBE2D3	MANBA|UBE2D3|CISD2|SLC9B1|SLC9B2
rs6564681	DYNLRB2|WWOX	NA	NA
rs6589706	DDX6	CXCR5	CXCR5
rs6589939	UBASH3B	UBASH3B	UBASH3B
rs6670198	TNFRSF14|UBE2D3	TNFRSF14|C1orf93	TNFRSF14|FAM213B|MMEL1|TTC34
rs6672420	RUNX3|SYF2	RUNX3	RUNX3
rs67111717	LMAN2	RGS14	LMAN2|RGS14|SLC34A1
rs6738544	NA|STAT4	STAT4	STAT4
rs6742	ZGPAT	NA	ZGPAT|NA|LIME1|NA|SLC2A4RG|ZBTB46
rs6789653	ZBTB38	RASA2|ZBTB38	NA

<table>
<tr><td>rs67934705</td><td>NA</td><td>NA</td><td>NA</td></tr>
<tr><td>rs6837324</td><td>TEC|TXK</td><td>TXK|TEC</td><td>TXK|TEC</td></tr>
<tr><td>rs6911131</td><td>FUCA2|LOC285740</td><td>PHACTR2</td><td>PHACTR2</td></tr>
<tr><td>rs6990534</td><td>LINC00977|MIR1208|PCAT1|POU5F1B</td><td>PVT1</td><td>NA</td></tr>
<tr><td>rs701006</td><td>TSFM</td><td>CYP27B1|MARCH9|METTL21B|B4GALNT1|OS9</td><td>DTX3|ARHGEF25|SLC26A10|B4GALNT1|OS9|AGAP2|AGAP2-AS1|TSPAN31|CDK4|MARCH9|CYP27B1|METTL1|METTL21B|NA|TSFM|AVIL|CTDSP2</td></tr>
<tr><td>rs719316</td><td>ATXN1</td><td>ATXN1</td><td>ATXN1</td></tr>
<tr><td>rs7222450</td><td>MAP3K14</td><td>NA</td><td>NA</td></tr>
<tr><td>rs7260482</td><td>PVR</td><td>PVR</td><td>IGSF23|PVR|CEACAM19</td></tr>
<tr><td>rs72922276</td><td>JAK1</td><td>JAK1</td><td>RAVER2|JAK1</td></tr>
<tr><td>rs72928038</td><td>BACH2</td><td>BACH2</td><td>BACH2</td></tr>
<tr><td>rs72989863</td><td>MARCH1</td><td>MARCH1</td><td>MARCH1</td></tr>
<tr><td>rs73414214</td><td>SYPL1</td><td>NA</td><td>SYPL1</td></tr>
<tr><td>rs735542</td><td>LINC00977|MIR1208|PCAT1|POU5F1B</td><td>NA</td><td>NA</td></tr>
<tr><td>rs760517</td><td>CSF2RB|NCF4</td><td>NCF4</td><td>NCF4</td></tr>
<tr><td>rs7731626</td><td>ANKRD55|NA</td><td>NA</td><td>ANKRD55</td></tr>
<tr><td>rs7855251</td><td>NANS</td><td>NA</td><td>TRIM14</td></tr>
<tr><td>rs7975763</td><td>MPHOSPH9</td><td>RILPL2|PITPNM2</td><td>ABCB9|OGFOD2|ARL6IP4|PITPNM2|MPHOSPH9|C12orf65|CDK2AP1|SBNO1|SETD8|RILPL2</td></tr>
<tr><td>rs7977720</td><td>CLEC2D|CLECL1</td><td>CLECL1|CLEC2D</td><td>CLEC2D|CLECL1</td></tr>
<tr><td>rs802730</td><td>NA</td><td>NA</td><td>PTPRK</td></tr>
<tr><td>rs8062446</td><td>NLRC5</td><td>NLRC5</td><td>NLRC5</td></tr>
<tr><td>rs883871</td><td>GSDMB</td><td>NA</td><td>THRA|NR1D1</td></tr>
<tr><td>rs9308424</td><td>BATF3|UBE2D3</td><td>BATF3</td><td>BATF3</td></tr>
<tr><td>rs9568402</td><td>DLEU7|ST13P4</td><td>NA</td><td>DLEU1</td></tr>
<tr><td>rs9591325</td><td>DLEU7|ST13P4</td><td>NA</td><td>DLEU1</td></tr>
<tr><td>rs9610458</td><td>MAPK1</td><td>PPM1F</td><td>YPEL1|MAPK1|PPM1F|TOP3B</td></tr>
<tr><td>rs962052</td><td>NA|RBM43|RND3</td><td>NA</td><td>NA</td></tr>
<tr><td>rs9808753</td><td>IL10RB</td><td>IFNGR2</td><td>IFNGR2|TMEM50B</td></tr>
<tr><td>rs983494</td><td>CD48|VANGL2</td><td>SLAMF7</td><td>SLAMF7</td></tr>
<tr><td>rs9843355</td><td>ARHGAP31</td><td>CD80|ARHGAP31|POGLUT1|TMEM39A</td><td>ARHGAP31|TMEM39A|POGLUT1|TIMMDC1|CD80</td></tr>
<tr><td>rs9863496</td><td>KCNH8|SATB1</td><td>SATB1</td><td>NA</td></tr>
<tr><td>rs9878602</td><td>FOXP1</td><td>FOXP1</td><td>FOXP1</td></tr>
<tr><td>rs9900529</td><td>GRB2</td><td>GRB2|GGA3|MIF4GD|SLC25A19|TSEN54</td><td>SLC25A19|GRB2</td></tr>
<tr><td>rs9909593</td><td>GSDMB</td><td>GSDMB|IKZF3|ORMDL3</td><td>GRB7|IKZF3|ZPBP2|GSDMB|ORMDL3|LRRC3C</td></tr>
<tr><td>rs9955954</td><td>MALT1</td><td>MALT1</td><td>MALT1</td></tr>
<tr><td>rs9992763</td><td>LEF1|RPL34-AS1</td><td>LEF1</td><td>LEF1</td></tr>
</table>

Supplementary Table 3. RegulomeDB content

Data Type	Types	Features
Transcription Factor ChIP-seq (ENCODE)	495 conditions/cell lines	7,721,822
Transcription Factor ChIP-seq (non-ENCODE)	32 conditions/cell lines	397,534
Transcription Factor ChIP-exo	1 condition	35,161
Histone Modifications	284 conditions/cell lines/marks	23,055,241
Dnase I hypersensitive sites	114 conditions/cell lines	20,710,098
FAIRE sites	25 conditions/cell lines	4,816,196
Dnase I footprints	50 cell lines	128,266,803
Predicted binding (PWMs)	1158 motifs	239,713,973
eQTLs	142,945 SNPs	142,945
dsQTLs	6069 SNPs	6,069
Manual annotations	6 genomic regions	282
VISTA enhancers	1448 enhancers	1,325
Validated SNPs affecting binding	855 SNPs	855

**Key**	
Data Type	Type of regulatory feature hosted by RegulomeDB
Types	Detailed content for each data type
Features	Total amount of regulatory features for data type

Supplementary Table 4. ENCODE cell types used in this analysis

cell_Line_name	ENCODE_Tier	Description	Tissue	Karyotype
GM13976	3	lymphoblastoid cell line, clinically normal; monozygotic twin sister with Cornelia De Lange syndrome is GM13977	blood	normal
GM12878	1	B-lymphocyte, lymphoblastoid, International HapMap Project - CEPH/Utah - European Caucasion, Epstein-Barr Virus	blood	normal
CD20+	2	B cells from donors RO01778 and RO01794, newly promoted to tier 2: not in 2011 analysis	blood	normal
CD20+_RO01778	2	B cells, caucasian, draw number 1, newly promoted to tier 2: not in 2011 analysis	blood	normal
CD20+_RO01794	2	B cells, African American, draw number 1, newly promoted to tier 2: not in 2011 analysis	blood	normal
GM10248	3	lymphoblastoid cell line, Clinically normal; 4 paternal cousins have Cornelia de Lange syndrome; 46,XY, t(3;22)(q25.3;p12)	blood	normal
GM12878-XiMat	3	B-lymphocyte, lymphoblastoid, cloned for maternal X inactivation, International HapMap Project, CEPH/Utah, Epstein-Barr Virus	blood	normal
GM12871	3	B-lymphocyte, lymphoblastoid, International HapMap Project, CEPH/Utah pedigree 1459, treatment: Epstein-Barr Virus transformed.	blood	normal
GM12872	3	B-lymphocyte, lymphoblastoid, International HapMap Project, CEPH/Utah pedigree 1459, treatment: Epstein-Barr Virus transformed	blood	normal
GM18507	3	lymphoblastoid, International HapMap Project, Yoruba in Ibadan, Nigera, treatment: Epstein-Barr Virus transformed	blood	normal
GM06990	3	B-lymphocyte, lymphoblastoid, International HapMap Project, CEPH/Utah, treatment: Epstein-Barr Virus transformed	blood	normal
GM10847	3	lymphoblastoid, International HapMap Project, CEPH/Utah, treatment: Epstein-Barr Virus transformed	blood	normal
GM12801	3	B-lymphocyte, lymphoblastoid, International HapMap Project, CEPH/Utah, treatment: Epstein-Barr Virus transformed	blood	normal
GM12812	3	B-Lymphocyte, Lymphoblastoid, International HapMap Project, CEPH/Utah, Treatment: Epstein-Barr Virus transformed	blood	normal
GM12813	3	B-Lymphocyte, Lymphoblastoid, International HapMap Project, CEPH/Utah, Treatment: Epstein-Barr Virus transformed	blood	normal
GM12864	3	B-lymphocyte, lymphoblastoid, International HapMap Project, CEPH/Utah pedigree 1459, treatment: Epstein-Barr Virus transformed	blood	normal
GM12865	3	B-lymphocyte, lymphoblastoid, International HapMap Project, CEPH/Utah pedigree 1459, treatment: Epstein-Barr Virus transformed	blood	normal
GM12866	3	B-lymphocyte, lymphoblastoid, International HapMap Project, CEPH/Utah pedigree 1459, treatment: Epstein-Barr Virus transformed.	blood	normal
GM12867	3	B-lymphocyte, lymphoblastoid, International HapMap Project, CEPH/Utah pedigree 1459, treatment: Epstein-Barr Virus transformed	blood	normal
GM12868	3	B-lymphocyte, lymphoblastoid, International HapMap Project, CEPH/Utah pedigree 1459, treatment: Epstein-Barr Virus transformed.	blood	normal
GM12869	3	B-lymphocyte, lymphoblastoid, International HapMap Project, CEPH/Utah pedigree 1459, treatment: Epstein-Barr Virus transformed.	blood	normal
GM12870	3	B-lymphocyte, lymphoblastoid, International HapMap Project, CEPH/Utah pedigree 1459, treatment: Epstein-Barr Virus transformed.	blood	normal
GM12873	3	B-lymphocyte, lymphoblastoid, International HapMap Project, CEPH/Utah pedigree 1459, treatment: Epstein-Barr Virus transformed	blood	normal
GM12874	3	B-lymphocyte, lymphoblastoid, International HapMap Project, CEPH/Utah pedigree 1459, treatment: Epstein-Barr Virus transformed	blood	normal
GM12875	3	B-lymphocyte, lymphoblastoid, International HapMap Project, CEPH/Utah pedigree 1459, treatment: Epstein-Barr Virus transformed	blood	normal
GM12891	3	B-lymphocyte, lymphoblastoid, International HapMap Project, CEPH/Utah pedigree 1463, treatment: Epstein-Barr Virus transformed	blood	normal
GM12892	3	B-lymphocyte, lymphoblastoid, International HapMap Project, CEPH/Utah pedigree 1463, treatment: Epstein-Barr Virus transformed	blood	normal
GM15510	3	lymphoblastoid NIGMS Human Genetic Cell Repository, DNA Polymorphism Discovery Resource Collection, treatment: Epstein-Barr Virus transformed	blood	normal
GM18505	3	lymphoblastoid, International HapMap Project, Yoruba in Ibadan, Nigera, treatment: Epstein-Barr Virus transformed	blood	normal
GM18526	3	lymphoblastoid, International HapMap Project, Han Chinese in Beijing, China, treatment: Epstein-Barr Virus transformed	blood	normal
PBMC	3	peripheral blood mononuclear cells	blood	normal
GM19099	3	lymphoblastoid, International HapMap Project, Yoruba in Ibadan, Nigera, treatment: Epstein-Barr Virus transformed	blood	normal
GM19193	3	lymphoblastoid, International HapMap Project, Yoruba in Ibadan, Nigera, treatment: Epstein-Barr Virus transformed	blood	normal
GM19238	3	B-lymphocyte, lymphoblastoid, International HapMap Project, Yoruba in Ibadan, Nigera, treatment: Epstein-Barr Virus transformed	blood	normal
GM18951	3	lymphoblastoid, International HapMap Project, Japanese in Tokyo, Japan, treatment: Epstein-Barr Virus transformed	blood	normal
H0287	3	EBV-transformed lymphoblastoid cell line (LCL) was derived from peripheral blood donated by a normal healthy male.	blood	normal
Lymphoblastoid	3	parental cell type to lymphoblastoid cell lines	blood	normal
Lymphoblastoid_cell_line	3	parental cell type to lymphoblastoid cell lines	blood	normal
H0287	3	EBV-transformed lymphoblastoid cell line (LCL) was derived from peripheral blood donated by a normal healthy male.	blood	normal
GM19239	3	B-lymphocyte, lymphoblastoid, International HapMap Project, Yoruba in Ibadan, Nigera, treatment: Epstein-Barr Virus transformed	blood	normal
GM19240	3	B-lymphocyte, lymphoblastoid, International HapMap Project, Yoruba in Ibadan, Nigera, treatment: Epstein-Barr Virus transformed	blood	normal
H0287	3	EBV-transformed lymphoblastoid cell line (LCL) was derived from peripheral blood donated by a normal healthy male.	blood	normal
Frontal_cortex_OC	3	Primary ventromedial prefrontal cortex, from KPBBB donor IDs 673 (Rep B1) and 913 (Rep B2)	frontal cortex	normal
H1-neurons	2	neurons derived from H1 embryonic stem cells, newly promoted to tier 2: not in 2011 analysis	neurons	normal
HAc	3	astrocytes-cerebellar	cerebellar	normal
NH-A	3	astrocytes (also called Astrocy)	brain	normal
Pons_OC	3	Primary frozen pons mid-brain tissue from NICHD donor IDs 1104 (Rep B1), 602 (Rep B2), 1442 (Rep B3)	pons mid-brain	normal
BC_Brain_H11058N	3	brain, donor H11058N, age 66, Asian, DNA extract	brain	normal
Cerebellum_OC	3	Primary frozen cerebellum tissue from NICHD donor IDs 1104 (Rep B1), 602 (Rep B2), 1442 (Rep B3), all Caucasian	cerebellum	normal
Cerebrum_frontal_OC	3	Primary frozen frontal cerebrum tissue from NICHD donor IDs 1104 (Rep B1), 602 (Rep B2), 1442 (Rep B3), all Caucasian	frontal cerebrum	normal
HA-sp	3	astrocytes spinal cord	spinal cord	normal
HA-h	3	astrocytes-hippocampal	brain hippocampus	normal
IMR90	2	fetal lung fibroblasts, newly promoted to tier 2: not in 2011 analysis	lung	normal
AG04450	3	fetal lung fibroblast	lung	normal
BC_Lung_01-11002	3	lung, donor 01-11002, age 83, caucasian, DNA and RNA extract	lung	normal
BC_Lung_H12817N	3	lung, donor H12817N, age 71, caucasian, DNA and RNA extract	lung	normal
HPF	3	pulmonary fibroblasts isolated from lung tissue	lung	normal
Lung_BC	3	lung, parental cell type to donors 01-11002 and H12817N	lung	normal
Lung_OC	3	Primary frozen lung tissue from NICHD donor IDs 1104 (Rep B1 DNase), 602 (Rep B2 DNase), 1442 (Rep B3 DNase) and CF Center donor IDs DD006I (Rep B1 FAIRE) and DD007I (Rep B2 FAIRE)	Lung	normal
NHLF	3	lung fibroblasts	lung	normal
WI-38	3	embryonic lung fibroblast cells, hTERT immortalized, includes Raf1 construct	embryonic lung	normal
FibroP_AG08396	3	fibroblasts taken from individuals with Parkinson's disease	lung fibroblast	normal
hMNC-CB	3	Mononuclear Cells (umbilical cord blood-single donor) from two individuals, hMNC-CB_9111701.6 and hMNC-CB_8072802.6	blood	normal
hMNC-CB_8072802.6	3	Mononuclear Cells, umbilical cord blood-single donor	blood	normal

Monocytes-CD14+	2	Monocytes-CD14+ are CD14-positive cells from human leukapheresis production, from donor RO 01746 (draw 1 ID is RO 01746, draw 2 ID is RO 01826), newly promoted to tier 2: not in 2011 analysis	monocytes	normal
Monocytes-CD14+_RO01746	2	Monocytes-CD14+ are CD14-positive cells from human leukapheresis production, from donor RO 01746 (draw 1 ID is RO 01746, draw 2 ID is RO 01826), Monocytes-CD14+_RO01746 and Monocytes-CD14+_RO01826 are being used as replicates, newly promoted to tier 2: not in 2011 analysis	monocytes	normal
Monocytes-CD14+_RO01826	2	Monocytes-CD14+ are CD14-positive cells from human leukapheresis production, Monocytes-CD14+_RO01826 is a second draw being used as a replicate for Monocytes-CD14+_RO01746, newly promoted to tier 2: not in 2011 analysis	monocytes	normal
GC_B_cell	3	Germinal center B cells (CD77+) isolated from de-identified tonsillectomy	tonsil	normal
BC_Stomach_H12817N	3	stomach, donor H12817N, age 71, caucasian, DNA and RNA extract	stomach	normal
Endometrium_OC	3	Endometrium isolated from uterine lining	endometrium	normal
Fibrobl_GM03348	3	skin fibroblast	skin	normal
FibroP	3	fibroblasts taken from individuals with Parkinson's disease, AG20443, AG08395 and AG08396 were pooled for this sample	skin	normal
GM04503	3	adult twin pair fibroblasts, monozygotic twin of GM04504	skin	normal
GM04504	3	adult twin pair fibroblasts, monozygotic twin of GM04503, 13% of the cells examined show random chromosome loss	skin	normal
HFF-Myc	3	foreskin fibroblast cells expressing canine cMyc	foreskin	normal
HGF	3	gingival fibroblasts	gingiva	normal
HIPEpiC	3	iris pigment epithelial cells	epithelium	normal
HWP	3	Undifferentiated White Preadipocytes from two individuals, HWP_0092205 and HWP_8120201.5, subcutaneous adipose tissue from abdomen / upper arm	adipose	normal
HWP_0092205	3	Undifferentiated White Preadipocytes from abdomen	subcutaneous adipose tissue	normal
Melano	3	epidermal melanocytes	skin	normal
FibroP_AG20443	3	fibroblasts taken from individuals with Parkinson's disease	skin fibroblast	normal
HFL11W	3	fetal liver 11 weeks, consented fetal liver samples were isolated from legally aborted fetuses at 11 weeks gestation	liver	normal
HFL24W	3	fetal liver 24 weeks, consented fetal liver samples were isolated from legally aborted fetuses at 24 weeks gestation	liver	normal
HHSEC	3	hepatic sinusoidal endothelial cells	liver	normal
iPS_CWRU1	3	iPS cells derived from MSC658 fibroblast	induced pluripotent cell (iPS)	normal
HMVEC-LBl	3	blood microvascular endothelial cells, lung-derived	blood vessel	normal
HMVEC-LLy	3	lymphatic microvascular endothelial cells, lung-derived	blood vessel	normal
TBEC	3	trachea and bronchial epithelial cells from frush lung tissue provided by National Disease Research Interchange, passage 2 primary, non-immortalized cells, donor was 21 years old	epithelium	normal
BC_Testis_N30	3	testis, donor N30, age 41, Asian, DNA extract	testis	normal
HMEC	3	mammary epithelial cells	breast	normal
HMEpC	3	Mammary Epithelial Cells (placeholder, waiting on second lot/donor from PromoCell)	mammary gland	normal
HMEpC_6022801.3	3	Mammary Epithelial Cells	mammary gland	normal
Myometr	3	myometrial cells	myometrium	normal
HMF	3	mammary fibroblasts	mammary	normal
PBMC	3	peripheral blood mononuclear cells	blood	normal
HBVP	3	brain vascular pericytes	blood vessel	normal
BC_Bladder_01-11002	3	urinary bladder, donor 01-11002, age 83, caucasian, DNA extract	urinary bladder	normal
BC_Uterus_BN0765	3	uterus, donor BN0765, age 44, Asian, DNA extract	uterus	normal
BJ	3	skin fibroblast, "The line was established from skin taken from normal foreskin." - ATCC. (PMID: 9916803)	skin	normal
HAEpiC	3	amniotic epithelial cells	epithelium	normal
BC_Leukocyte_UHN00204	3	peripheral blood mononuclear cell, donor UHN00204, age 54, caucasian, DNA extract	blood	normal
hMNC-CB_9111701.6	3	Mononuclear Cells, umbilical cord blood-single donor	blood	normal
hMNC-PB	3	Mononuclear Cells (peripheral blood-single donor) from two individuals, hMNC-PB_0022330.9 and hMNC-PB_0082430.9	blood	normal
hMNC-PB_0022330.9	3	Mononuclear Cells, peripheral blood-single donor	blood	normal
hMNC-PB_0082430.9	3	Mononuclear Cells, peripheral blood-single donor	blood	normal
Naive_B_cell	3	Naive B cells (IgD+) isolated from de-identified tonsillectomy	tonsil	normal
HBMEC	3	brain microvascular endothelial cells	blood vessel	normal
HBVSMC	3	brain vascular smooth muscle cells.	blood vessel	normal
HCPEpiC	3	choroid plexus epithelial cells	epithelium	normal
BC_Leukocyte_UHN00204	3	peripheral blood mononuclear cell, donor UHN00204, age 54, caucasian, DNA extract	blood	normal
hMNC-CB	3	Mononuclear Cells (umbilical cord blood-single donor) from two individuals, hMNC-CB_9111701.6 and hMNC-CB_8072802.6	blood	normal
hMNC-CB_8072802.6	3	Mononuclear Cells, umbilical cord blood-single donor	blood	normal
hMNC-CB_9111701.6	3	Mononuclear Cells, umbilical cord blood-single donor	blood	normal
hMNC-PB	3	Mononuclear Cells (peripheral blood-single donor) from two individuals, hMNC-PB_0022330.9 and hMNC-PB_0082430.9	blood	normal
hMNC-PB_0022330.9	3	Mononuclear Cells, peripheral blood-single donor	blood	normal
hMNC-PB_0082430.9	3	Mononuclear Cells, peripheral blood-single donor	blood	normal
HUVEC	2	umbilical vein endothelial cells	blood vessel	normal
AG04449	3	fetal buttock/thigh fibroblast	skin	normal
AG09319	3	gum tissue fibroblasts from apparently heathly 24 year old	gingival	normal
AoAF	3	aortic adventitial fibroblast cells	blood vessel	normal
AoSMC	3	aortic smooth muscle cells	blood vessel	normal
BC_Adipose_UHN00001	3	adipose tissue, donor UHN0001, age 35, African American, DNA extract	adipose tissue	normal
BC_Adrenal_Gland_H12803N	3	adrenal gland, donor H12803N, age 88, caucasian, DNA extract	adrenal gland	normal
BC_Breast_02-03015	3	breast, donor 02-03015, age 21, caucasian, DNA extract	breast	normal
BC_Colon_01-11002	3	colon, donor 01-11002, age 83, caucasian, DNA and RNA extract	colon	normal
BC_Colon_H12817N	3	colon, donor H12817N, age 71, caucasian, DNA and RNA extract	colon	normal

BC_Esophagus_01-11002	3	esophagus, donor 01-11002, age 83, caucasian, DNA and RNA extract	esophagus	normal
BC_Esophagus_H12817N	3	esophagus, donor H12817N, age 71, caucasian, DNA extract	esophagus	normal
BC_Jejunum_H12817N	3	jejunum, donor H12817N, age 71, caucasian, DNA and RNA extract	jejunum	normal
BC_Kidney_01-11002	3	kidney, donor 01-11002, age 83, caucasian, DNA and RNA extract	kidney	normal
BC_Kidney_H12817N	3	kidney, donor H12817N, age 71, caucasian, DNA and RNA extract	kidney	normal
BC_Left_Ventricle_N41	3	left ventricle, donor N41, age 26, Asian, DNA extract	left ventricle	normal
BC_Liver_01-11002	3	liver, donor 01-11002, age 83, caucasian, DNA and RNA extract	liver	normal
BC_Pancreas_H12817N	3	pancreas, donor H12817N, age 71, caucasian, DNA and RNA extract	pancreas	normal
BC_Penis_H12817N	3	penis, donor H12817N, age 71, caucasian, DNA extract	penis	normal
BC_Pericardium_H12529N	3	pericardium, donor H12529N, age 70, caucasian, DNA extract	pericardium	normal
BC_Placenta_UHN00189	3	placenta, donor UHN00189, age 29	placenta	normal
BC_Prostate_Gland_H12817N	3	prostate gland, donor H12817N, age 71, caucasian, DNA extract	prostate gland	normal
BC_Rectum_N29	3	rectum, donor N29, age 29, Asian, DNA extract	rectum	normal
BC_Skeletal_Muscle_01-11002	3	skeletal muscle, donor 01-11002, age 83, caucasian, DNA and RNA extract	skeletal muscle	normal
BC_Skeletal_Muscle_H12817N	3	skeletal muscle, donor H12817N, age 71, caucasian, DNA and RNA extract	skeletal muscle	normal
BC_Skin_01-11002	3	skin, donor 01-11002, age 83, caucasian, DNA extract	skin	normal
BC_Small_Intestine_01-11002	3	small intestine, donor 01-11002, age 83, caucasian, DNA and RNA extract	small intestine	normal
BC_Spleen_H12817N	3	spleen, donor H12817N, age 71, caucasian, DNA and RNA extract	spleen	normal
BC_Stomach_01-11002	3	stomach, donor 01-11002, age 83, caucasian, DNA extract	stomach	normal
bone_marrow_HS27a	3	HS27a Human Marrow Stromal Cells are fibroblastoid cells immortalized with HPV16 E6/E7 genes as described in Roecklein and Torok-Storb, 1995 Blood 85:997-1005, insert DNA is LXSN-16 E6E7 packaged in PA31.	bone marrow	normal
bone_marrow_HS5	3	HS5 Human Marrow Stromal Cells are fibroblastoid cells immortalized with HPV16 E6/E7 genes as described in Roecklein and Torok-Storb, 1995 Blood 85:997-1005, insert DNA is LXSN-16 E6E7 packaged in PA31.	bone marrow	normal
bone_marrow_MSC	3	These cells are primary fibroblastoid cells obtained from human bone marrow of normal donors as described in Roecklein and Torok-Storb, 1995 Blood 85:997-1005.	bone marrow	normal
Breast_OC	3	Primary frozen breast tissue from NCTC donor IDs 11-0068A (Rep B1) and 11-0067A (Rep B2), both African American	breast	normal
Colon_BC	3	colon, parent cell type to donors BC_01-11002 and BC_H12817N	colon	normal
Colon_OC	3	Primary frozen colon tissue from NCTC donor IDs 10-0005A (Rep B1) and 10-0170A (Rep B2), African American and caucasian	colon	normal
Esophagus_BC	3	esophagus, parental cell type to donors 01-11002 and H12817N	esophagus	normal
Fibrobl	3	child fibroblast	skin	normal
HAoAF	3	Aortic Adventitial Fibroblasts from two individuals, HAoAF_6090101.11 and HAoAF_6111301.9 from tunica adventitia	blood vessel	normal
HAoAF_6090101.11	3	Aortic Adventitial Fibroblasts from tunica adventitia	blood vessel	normal
HAoAF_6111301.9	3	Aortic Adventitial Fibroblasts from tunica adventitia	blood vessel	normal
HAoEC	3	Aortic Endothelial Cells (thoracic) from two individuals, HAoEC_7071706.1 and HAoEC_8061102.1	blood vessel	normal
HAoEC_7071706.1	3	Aortic Endothelial Cells	blood vessel	normal
HAoEC_8061102.1	3	Aortic Endothelial Cells from throracic	blood vessel	normal
HCF	3	cardiac fibroblasts	heart	normal
HCFaa	3	cardiac fibroblasts- adult atrial	heart	normal
HCH	3	Undifferentiated Chondrocytes from two individuals, HCH_8100808.2 and HCH_0011308.2P from knee joint	cartilage	normal
HCH_0011308.2P	3	Undifferentiated Chondrocytes from knee joint	cartilage	normal
HCH_8100808.2	3	Undifferentiated Chondrocytes from knee joint	cartilage	normal
HCM	3	cardiac myocytes	heart	normal
Heart_OC	3	Primary frozen heart tissue from NICHD donor IDs 1104 (Rep B1 DNase), 602 (Rep B2 DNase), 1442 (Rep B3 DNase), 1863 (Rep B1 FAIRE), 4548 (Rep B2 FAIRE)	heart	normal
Heart_STL003	3	Standard procurement of heart tissue from a 34 year old caucasian male.	heart	normal
HEEpiC	3	esophageal epithelial cells	epithelium	normal
Hepatocytes	3	primary hepatocytes, liver perfused by enzymes to generate single cell suspension	liver	normal
HFDPC	3	Follicle Dermal Papilla Cells from two individuals, HFDPC_0100503.2 and HFDPC_0102703.3 from lateral scalp (brown, blond)	skin	normal
HFDPC_0100503.2	3	Follicle Dermal Papilla Cells from lateral scalp (brown)	skin	normal
HFDPC_0102703.3	3	Follicle Dermal Papilla Cells from lateral scalp (blond)	skin	normal
HFF	3	foreskin fibroblast	foreskin	normal
hMSC-AT	3	Undifferentiated Mesenchymal Stem Cells from two individuals, hMSC-AT_9061601.12 and hMSC-AT_0102604.12 from subcutaneous abdomen adipose tissue	adipose	normal
hMSC-AT_0102604.12	3	Undifferentiated Mesenchymal Stem Cells from subcutaneous abdomen adipose tissue	adipose	normal
hMSC-AT_9061601.12	3	Undifferentiated Mesenchymal Stem Cells from abdomen	subcutaneous adipose tissue	normal
hMSC-BM	3	Undifferentiated Mesenchymal Stem Cells from two individuals, hMSC-BM_0050602.11 and hMSC-BM_0051105.11 from femoral head	bone marrow	normal
hMSC-BM_0050602.11	3	Undifferentiated Mesenchymal Stem Cells from femoral head	bone marrow	normal
hMSC-BM_0051105.11	3	Undifferentiated Mesenchymal Stem Cells from femoral head	bone marrow	normal
hMSC-UC	3	Undifferentiated Mesenchymal Stem Cells from two individuals, hMSC-UC_0081101.7 and hMSC-UC_0052501.7 from matrix (Wharton's Jelly)	umbilical cord	normal
hMSC-UC_0052501.7	3	Undifferentiated Mesenchymal Stem Cells from matrix (Wharton's Jelly)	umbilical cord	normal
hMSC-UC_0081101.7	3	Undifferentiated Mesenchymal Stem Cells from matrix (Wharton's Jelly)	umbilical cord	normal
HMVEC-dAd	3	adult dermal microvascular endothelial cells.	blood vessel	normal
HMVEC-dBl-Ad	3	adult blood microvascular endothelial cells, dermal-derived	blood vessel	normal
HMVEC-dBl-Neo	3	neonatal blood microvascular endothelial cells, dermal-derived	blood vessel	normal
HMVEC-dLy-Ad	3	adult lymphatic microvascular endothelial cells, dermal-derived	blood vessel	normal
HMVEC-dLy-Neo	3	neonatal lymphatic microvascular endothelial cells, dermal-derived	blood vessel	normal

HMVEC-dNeo	3	neonatal microvascular endothelial cells (single donor), dermal-derived	blood vessel	normal
HNPCEpiC	3	non-pigment ciliary epithelial cells	epithelium	normal
HOB	3	Undifferentiated Osteoblasts from two individuals, HOB_0090202.1 and HOB_0091301 from femoral head	cancellous bone	normal
HOB_0090202.1	3	Undifferentiated Osteoblasts from femoral head	cancellous bone	normal
HOB_0091301	3	Undifferentiated Osteoblasts from femoral head	cancellous bone	normal
HPAEC	3	pulmonary artery endothelial cells.	blood vessel	normal
HPAEpiC	3	pulmonary alveolar epithelial cells	epithelium	normal
HPAF	3	pulmonary artery fibroblasts	blood vessel	normal
HPDE6-E6E7	3	pancreatic duct cells immortalized with E6E7 gene of HPV	pancreatic duct	normal
HPdLF	3	periodontal ligament fibroblasts	epithelium	normal
HPIEpC	3	Placental Epithelial Cells amniotic membrane	placenta	normal
HPIEpC_9012801.2	3	Placental Epithelial Cells amniotic membrane	placenta	normal
HPIEpC_9041503.2	3	Placental Epithelial Cells amniotic membrane	placenta	normal
HRCEpiC	3	renal cortical epithelial cells	epithelium	normal
HRE	3	renal epithelial cells	epithelium	normal
HRGEC	3	renal glomerular endothelial cells	kidney	normal
HRPEpiC	3	retinal pigment epithelial cells	epithelium	normal
HSaVEC	3	Saphenous Vein Endothelial Cells from two individuals, HSaVEC_9100101.15 and HSaVEC_0022202.16 from thigh	blood vessel	normal
HSaVEC_0022202.16	3	Saphenous Vein Endothelial Cells from thigh	blood vessel	normal
HSaVEC_9100101.15	3	Saphenous Vein Endothelial Cells from thigh	blood vessel	normal
HSMM	3	skeletal muscle myoblasts	muscle	normal
HSMMtube	3	skeletal muscle myotubes differentiated from the HSMM cell line	muscle	normal
HTR8svn	3	trophoblast (HTR-8/SVneo) cell line, a thin layer of ectoderm that forms the wall of many mammalian blastulas and functions in the nutrition and implantation of the embryo	blastula	normal
HVMF	3	villous mesenchymal fibroblast cells	connective	normal
HVMF_6091203.3	3	Villous Mesenchymal Fibroblasts from villous tissue	placenta	normal
HVMF_6100401.3	3	Villous Mesenchymal Fibroblasts from villous tissue	placenta	normal
HWP_8120201.5	3	Undifferentiated White Preadipocytes from upper arm	subcutaneous adipose tissue	normal
iPS_hFib2_iPS4	3	induced pluripotent stem cell line 4 derived from skin fibroblast of a 30 year old donor, same donor as iPS_hFib2_iPS5	induced pluripotent cell (iPS)	normal
iPS_hFib2_iPS5	3	induced pluripotent stem cell line 5 derived from skin fibroblast of a 30 year old donor, same donor as iPS_hFib2_iPS4	induced pluripotent cell (iPS)	normal
Kidney_BC	3	kidney, parental cell type to donors 01-11002 and H12817N	kidney	normal
Kidney_OC	3	Primary frozen kidney tissue from NICHD donor IDs 1104 (Rep B1 DNase), 602 (Rep B2 DNase), 1442 (Rep B3 DNase), 1863 (Rep B3 FAIRE) and NCTC donor ID 10-0022A (Rep B1 FAIRE)	kidney	normal
LHSR	3	prostate epithelial cells (PrEC), multiple human donors, all of whom are HIV-1, Hepatitis B and Hepatitis C negative, treatment: to create LHSR, cells were infected with amphotropic retroviruses encoding the SV40 large T antigen (L), the telomerase catalytic subunit hTERT (H), the SV40 small T antigen (S) and an oncogenic allele of H-ras (R).	epithelium	normal
Liver_OC	3	Primary frozen liver tissue from NICHD donor IDs 1104 (Rep B1), 602 (Rep B2), 1442 (Rep B3)	liver	normal
Liver_STL004	3	Primary liver tissue from the right portion of a standard liver procurement from a 6 year old female.	liver	normal
Liver_STL011	3	Primary liver tissue of a standard liver procurement from a 36 year old caucasian male.	liver	normal
NHBE	3	bronchial epithelial cells	epithelium	normal
NHBE_RA	3	bronchial epithelial cells with retinoic acid	bronchial epithelium	normal
NHDF	3	Dermal Fibroblasts from temple / breast	skin	normal
NHDF-Ad	3	adult dermal fibroblasts	skin	normal
NHDF-neo	3	neonatal dermal fibroblasts	skin	normal
NHDF_0060801.3	3	Dermal Fibroblasts from temple	skin	normal
NHDF_7071701.2	3	Dermal Fibroblasts from breast	skin	normal
NHEK	3	epidermal keratinocytes	skin	normal
NHEM.f_M2	3	Epidermal Melanocytes from two individuals, NHEM.f_M2_5071302.2 and NHEM.f_M2_6022001 from foreskin	skin	normal
NHEM.f_M2_5071302.2	3	Epidermal Melanocytes (foreskin)	skin	normal
NHEM.f_M2_6022001	3	Epidermal Melanocytes (foreskin)	skin	normal
NHEM_M2	3	Epidermal Melanocytes (adult) from two individuals, NHEM_M2_7011001.2 and NHEM_M2_7012303 from cheek / temple	skin	normal
NHEM_M2_7011001.2	3	Epidermal Melanocytes (adult) from cheek	skin	normal
NHEM_M2_7012303	3	Epidermal Melanocytes (adult) from temple	skin	normal
Olf_neurosphere	3	Human olfactory neurosphere-derived cells from mucosal biopsies	Nasal biopsy	normal
Osteobl	3	osteoblasts (NHOst)	bone	normal
Pancreas_OC	3	Primary frozen pancreas tissue from NCTC donor IDs 09-0144A (Rep B1) and 10-0021A (Rep B2)	pancreas	normal
PanIslets	3	pancreatic islets from 2 donors, the sources of these primary cells are cadavers from National Disease Research Interchange (NDRI) and another sample isolated as in Bucher, P. et al., Assessment of a novel two-component enzyme preparation for human islet isolation and transplantation. Transplantation 79, 917 (2005)	pancreas	normal
PBDEFetal	3	peripheral blood-derived erythroblasts from 16-19 week human fetal liver	liver	normal
PrEC	3	prostate epithelial cell line	prostate	normal
Prostate_OC	3	Primary frozen prostate tissue from NICHD donor IDs 1669 (Rep B1) and 1540 (Rep B2)	prostate	normal
Psoas_muscle_OC	3	Primary frozen psoas muscle tissue from NICHD donor IDs 1104 (Rep B1 DNase), 602 (Rep B2 DNase), 1442 (Rep B3 DNase), 1540 (Rep B1), 1863 (Rep B2 FAIRE)	psoas muscle	normal
RPTEC	3	renal proximal tubule epithelial cells	epithelium	normal
RWPE1	3	prostate epithelial	prostate	normal

SAEC	3	small airway epithelial cells	epithelium	normal
Skeletal_Muscle_BC	3	skeletal muscle, parental cell type to donors 01-11002 and H12817N	skeletal muscle	normal
SkMC	3	Skeletal Striated Muscle Cells from two individuals, SkMC_9011302 SkMC_8121902.17 from M. pectoralis / Mm. intercostales	muscle	normal
SKMC	3	skeletal muscle cells	muscle	normal
SkMC_8121902.17	3	Skeletal Muscle Cells from Mm. intercostales	striated muscle	normal
SkMC_9011302	3	Skeletal Muscle Cells from M. pectoralis	striated muscle	normal
Small_intestine_OC	3	Primary frozen small intestine tissue from NCTC donor IDs 10-0063A (Rep B1) and 09-0143A (Rep B2)	small intestine	normal
Spleen_OC	3	Primary frozen spleen tissue from NICHD donor IDs 1863 (Rep B1) and 4548 (Rep B2)	spleen	normal
Stellate	3	hepatic stellate cells, liver that was perfused with collagenase and sellected for hepatic stellate cells by density gradient	liver	normal
Stomach_BC	3	stomach, parental cell type to donors 01-11002 and H12817N	stomach	normal
Urothelia	3	primary ureter cell culture of urothelial cells derived from a 12 year-old girl and immortalized by transfection with a temperature-sensitive SV-40 large T antigen gene	urothelium	normal
Decidua	3	decidua cells (part of the mucous membrane lining uterus), Fetal membranes were collected from women who underwent planned cesarean delivery at term, before labor and without rupture of membranes	cervix	normal
HAL	3	adult liver, genomic DNA purified from surgically excised adult human liver	liver	normal
FibroP_AG08395	3	fibroblasts taken from individuals with Parkinson's disease	skin fibroblast	normal
CD34+_Mobilized	3	hematopoietic progenitor cells- mobilized, from donor RO01679.	blood	normal
CD34+_Mobilized	3	hematopoietic progenitor cells- mobilized, from donor RO01679.	blood	normal
LHCN-M2	2	skeletal myoblasts derived from satellite cells from the pectoralis major muscle of a 41 year old caucasian heart transplant donor, immortalized with lox-hygro-hTERT ("LH"), and Cdk4-neo ("CN"), Zhu et al. (2007) in Aging Cell, vol. 6, pp 515-523, newly promoted to tier 2: not in 2011 analysis.	skeletal muscle myoblast	normal
Chorion	3	chorion cells (outermost of two fetal membranes), fetal membranes were collected from women who underwent planned cesarean delivery at term, before labor and without rupture of membranes.	fetal membrane	normal
HConF	3	conjunctival fibroblast	eye	normal
HEK293	3	embryonic kidney, cells contain Adenovirus 5 DNA (PMID: 11967234)	kidney	normal
HEK293-T-REx	3	embryonic kidney cells transformed with Adenovirus 5 DNA stably expressing tetracycline repressor, HEK293 (ATCC number CRL-1573) is the parental cell line, hypotriploid, XXX	kidney	normal
HEK293T	3	embryonic kidney that expresses SV40 large T antigen, HEK293 (ATCC number CRL-1573) is the parental cell line	kidney	normal
HSMM_emb	3	embryonic myoblast	muscle	normal
HSMM_FSHD	3	primary myoblast from Facioscapulohumeral Muscular Dystrophy (FSHD) patients, muscle needle biopsies	muscle	normal
HSMMtube_emb	3	embryonic myotube	muscle	normal
HSMMtube_FSHD	3	myotube from Facioscapulohumeral Muscular Dystrophy (FSHD) patient, muscle needle biopsies	muscle	normal
iPS	3	induced pluripotent stem cell derived from skin fibroblast	induced pluripotent stem cell	normal
iPS_NIHi11	3	iPS cells derived from AG20443 fibroblast	induced pluripotent cell (iPS)	normal
iPS_NIHi7	3	iPS cells derived from AG08395 fibroblast	induced pluripotent cell (iPS)	normal
Ishikawa	3	endometrial adenocarcinoma	uterus	normal
MCF10A-Er-Src	3	mammary gland, non-tumorigenic epithelial, inducible cell line, derived from the MCF-10A parental cells and contain ER-Src, a derivative of the Src kinase oncoprotein (v-Src) that is fused to the ligand-binding domain of the estrogen receptor (ER)	breast	normal
PanIsletD	3	dedifferentiated human pancreatic islets from the National Disease Research Interchange (NDRI), same source as PanIslets	pancreas	normal
PBDE	3	peripheral blood-derived erythroblasts	blood	normal
pHTE	3	primary tracheal epithelial cells	epithelium	normal
ProgFib	3	fibroblasts, Hutchinson-Gilford progeria syndrome (cell line HGPS, HGADFN167, progeria research foundation)	skin	normal
prostate	3	prostate tissue purchased for CSHL project	prostate	normal
BC_Leukocyte_UHN00204	3	peripheral blood mononuclear cell, donor UHN00204, age 54, caucasian, DNA extract	blood	normal
hMNC-CB	3	Mononuclear Cells (umbilical cord blood-single donor) from two individuals, hMNC-CB_9111701.6 and hMNC-CB_8072802.6	blood	normal
hMNC-CB_8072802.6	3	Mononuclear Cells, umbilical cord blood-single donor	blood	normal
hMNC-CB_9111701.6	3	Mononuclear Cells, umbilical cord blood-single donor	blood	normal
hMNC-PB	3	Mononuclear Cells (peripheral blood-single donor) from two individuals, hMNC-PB_0022330.9 and hMNC-PB_0082430.9	blood	normal
hMNC-PB_0022330.9	3	Mononuclear Cells, peripheral blood-single donor	blood	normal
hMNC-PB_0082430.9	3	Mononuclear Cells, peripheral blood-single donor	blood	normal
CD34+_Mobilized	3	hematopoietic progenitor cells- mobilized, from donor RO01679.	blood	normal
PBMC	3	peripheral blood mononuclear cells	blood	normal
Adult_CD4_naive	3	CD4+ cells isolated from human blood and enriched for naive populations	blood	normal
Adult_CD4_Th0	3	CD4+ cells isolated from human blood and enriched for Th0 populations	blood	normal
CD4+_Naive_Wb11970640	3	CD4+ naive sorted cells, donor is Caucasian, male 26 year old, primary pheresis of single normal subject	blood	normal
CD4+_Naive_Wb78495824	3	CD4+ naive sorted cells, donor is Causasian, female 35 year old, primary pheresis of single normal subject	blood	normal
Cord_CD4_naive	3	CD4+ cells isolated from cord blood and enriched for naive populations	blood	normal
Cord_CD4_Th0	3	CD4+ cells isolated from cord blood and enriched for Th0 populations	blood	normal
Adult_CD4_Th1	3	CD4+ cells isolated from human blood and enriched for Th1 populations	blood	normal
Cord_CD4_Th1	3	CD4+ cells isolated from cord blood and enriched for Th1 populations	blood	normal
Th1_Wb54553204	3	Th1 cells in vivo isolation, donor is Caucasian, male 33 year old, primary pheresis of single normal subject	blood	normal
Th1	3	primary Th1 T cells	blood	normal
Th17	3	T helper cells expressing IL-17, primary pheresis of single normal subject	blood	normal
Th2_Wb33676984	3	Th2 cells in vivo isolation, donor is Asian, female 26 year old, primary pheresis of single normal subject	blood	normal
Th2_Wb54553204	3	Th2 cells in vivo isolation, donor is Caucasian, male 33 year old, primary pheresis of single normal subject	blood	normal
Th2	3	primary Th2 T cells	blood	normal
Treg_Wb83319432	3	T regulatory cells in vivo isolation, donor is Caucasian, male 28 year old, primary pheresis of single normal subject	blood	normal

Key	
cell_Line_name	Cell line name
ENCODE_Tier	ENCODE tier (phase)
Description	Description of ENCODE cell line
Tissue	Tissue or cell type
Karyotype	Karyotype (Normal or cancer)
Cell Bucket for analysis	Major cell type associated to cell line for this analysis
B:	B cells
T:	T cells
M:	Monocytes/macrophages
O:	Other
L:	Lung

Supplementary Table 5. Epigenomic Roadmap Project cells used in this analysis

Standardized Epigenome name	Epigenome name (from EDACC Release 9 directory)	ANATOMY	TYPE	Cell Bucket for Aanalysis
Primary B cells from cord blood	CD19_Primary_Cells_Cord_BI	BLOOD	PrimaryCell	B
Primary B cells from peripheral blood	CD19_Primary_Cells_Peripheral_UW	BLOOD	PrimaryCell	B
GM12878 Lymphoblastoid Cell Line	GM12878_Lymphoblastoid	BLOOD	CellLine	B
Brain Cingulate Gyrus	Brain_Cingulate_Gyrus	BRAIN	PrimaryTissue	C
Brain Dorsolateral Prefrontal Cortex	Brain_Dorsolateral_Prefrontal_Cortex	BRAIN	PrimaryTissue	C
Brain Germinal Matrix	Brain_Germinal_Matrix	BRAIN	PrimaryTissue	C
Brain Hippocampus Middle	Brain_Hippocampus_Middle	BRAIN	PrimaryTissue	C
Brain Inferior Temporal Lobe	Brain_Inferior_Temporal_Lobe	BRAIN	PrimaryTissue	C
Brain Substantia Nigra	Brain_Substantia_Nigra	BRAIN	PrimaryTissue	C
Cortex derived primary cultured neurospheres	Neurosphere_Cultured_Cells_Cortex_Derived	BRAIN	PrimaryCell	C
Fetal Brain Female	Fetal_Brain_Female	BRAIN	PrimaryTissue	C
Brain Angular Gyrus	Brain_Angular_Gyrus	BRAIN	PrimaryTissue	C
Brain Anterior Caudate	Brain_Anterior_Caudate	BRAIN	PrimaryTissue	C
Fetal Brain Male	Fetal_Brain_Male	BRAIN	PrimaryTissue	C
Ganglion Eminence derived primary cultured neurospheres	Neurosphere_Cultured_Cells_Ganglionic_Eminence_Derived	BRAIN	PrimaryCell	C
H9 Derived Neuronal Progenitor Cultured Cells	H9_Derived_Neuronal_Progenitor_Cultured_Cells	ESC_DERIVED	CellLineDerived	C
H1 Derived Neuronal Progenitor Cultured Cells	H1_Derived_Neuronal_Progenitor_Cultured_Cells	ESC_DERIVED	CellLineDerived	C
H9 Derived Neuron Cultured Cells	H9_Derived_Neuron_Cultured_Cells	ESC_DERIVED	CellLineDerived	C
H1 Derived Mesenchymal Stem Cells	H1_Derived_Mesenchymal_Stem_Cells	ESC_DERIVED	CellLineDerived	L
IMR90 fetal lung fibroblasts Cell Line	IMR90_Cell_Line	LUNG	CellLine	L
NHLF Lung Fibroblast Primary Cells	NHLF_Lung_Fibroblasts	LUNG	PrimaryCell	L
Fetal Lung	Fetal_Lung	LUNG	PrimaryTissue	L
Lung	Lung	LUNG	PrimaryTissue	L
Monocytes-CD14+ RO01746 Primary Cells	Monocytes-CD14+_RO01746	BLOOD	PrimaryCell	M
Primary monocytes from peripheral blood	CD14_Primary_Cells	BLOOD	PrimaryCell	M
Fetal Adrenal Gland	Fetal_Adrenal_Gland	ADRENAL	PrimaryTissue	O
Primary hematopoietic stem cells	CD34_Primary_Cells	BLOOD	PrimaryCell	O
Primary hematopoietic stem cells G-CSF-mobilized Female	Mobilized_CD34_Primary_Cells_Female	BLOOD	PrimaryCell	O
Primary hematopoietic stem cells G-CSF-mobilized Male	Mobilized_CD34_Primary_Cells_Male	BLOOD	PrimaryCell	O
Primary hematopoietic stem cells short term culture	CD34_Cultured_Cells	BLOOD	PrimaryCell	O
Primary mononuclear cells from peripheral blood	Peripheral_Blood_Mononuclear_Primary_Cells	BLOOD	PrimaryCell	O
Primary neutrophils from peripheral blood	CD15_Primary_Cells	BLOOD	PrimaryCell	O
Osteoblast Primary Cells	Osteoblasts	BONE	PrimaryCell	O
Breast Myoepithelial Primary Cells	Breast_Myoepithelial_Cells	BREAST	PrimaryCell	O
Breast variant Human Mammary Epithelial Cells (vHMEC)	Breast_vHMEC	BREAST	PrimaryCell	O
HMEC Mammary Epithelial Primary Cells	HMEC_Mammary_Epithelial	BREAST	PrimaryCell	O
HUES6 Cell Line	HUES6_Cell_Line	ESC	CellLine	O
ES-UCSF4 Cell Line	4star	ESC	CellLine	O
ES-I3 Cell Line	ES-I3_Cell_Line	ESC	CellLine	O
ES-WA7 Cell Line	ES-WA7_Cell_Line	ESC	CellLine	O
H1 Cell Line	H1_Cell_Line	ESC	CellLine	O
H9 Cell Line	H9_Cell_Line	ESC	CellLine	O
HUES48 Cell Line	HUES48_Cell_Line	ESC	CellLine	O
HUES64 Cell Line	HUES64_Cell_Line	ESC	CellLine	O
H1 BMP4 Derived Mesendoderm Cultured Cells	H1_BMP4_Derived_Mesendoderm_Cultured_Cells	ESC_DERIVED	CellLineDerived	O
H1 BMP4 Derived Trophoblast Cultured Cells	H1_BMP4_Derived_Trophoblast_Cultured_Cells	ESC_DERIVED	CellLineDerived	O
hESC Derived CD184+ Endoderm Cultured Cells	hESC_Derived_CD184+_Endoderm_Cultured_Cells	ESC_DERIVED	CellLineDerived	O
hESC Derived CD56+ Ectoderm Cultured Cells	hESC_Derived_CD56+_Ectoderm_Cultured_Cells	ESC_DERIVED	CellLineDerived	O
hESC Derived CD56+ Mesoderm Cultured Cells	hESC_Derived_CD56+_Mesoderm_Cultured_Cells	ESC_DERIVED	CellLineDerived	O
Adipose Derived Mesenchymal Stem Cell Cultured Cells	Adipose_Derived_Mesenchymal_Stem_Cell_Cultured_Cells	FAT	PrimaryCell	O
Adipose Nuclei	Adipose_Nuclei	FAT	PrimaryTissue	O
Mesenchymal Stem Cell Derived Adipocyte Cultured Cells	Mesenchymal_Stem_Cell_Derived_Adipocyte_Cultured_Cells	FAT	CellLineDerived	O
Colon Smooth Muscle	Colon_Smooth_Muscle	GI_COLON	PrimaryTissue	O
Colonic Mucosa	Colonic_Mucosa	GI_COLON	PrimaryTissue	O
Sigmoid Colon	Sigmoid_Colon	GI_COLON	PrimaryTissue	O
Duodenum Mucosa	Duodenum_Mucosa	GI_DUODENUM	PrimaryTissue	O
Duodenum Smooth Muscle	Duodenum_Smooth_Muscle	GI_DUODENUM	PrimaryTissue	O
Esophagus	Esophagus	GI_ESOPHAGUS	PrimaryTissue	O
Fetal Intestine Large	Fetal_Intestine_Large	GI_INTESTINE	PrimaryTissue	O
Fetal Intestine Small	Fetal_Intestine_Small	GI_INTESTINE	PrimaryTissue	O
Small Intestine	Small_Intestine	GI_INTESTINE	PrimaryTissue	O
Rectal Mucosa Donor 29	Rectal_Mucosa.Donor_29	GI_RECTUM	PrimaryTissue	O
Rectal Mucosa Donor 31	Rectal_Mucosa.Donor_31	GI_RECTUM	PrimaryTissue	O
Rectal Smooth Muscle	Rectal_Smooth_Muscle	GI_RECTUM	PrimaryTissue	O
Gastric	Gastric	GI_STOMACH	PrimaryTissue	O
Stomach Mucosa	Stomach_Mucosa	GI_STOMACH	PrimaryTissue	O
Stomach Smooth Muscle	Stomach_Smooth_Muscle	GI_STOMACH	PrimaryTissue	O
Fetal Heart	Fetal_Heart	HEART	PrimaryTissue	O
Left Ventricle	Left_Ventricle	HEART	PrimaryTissue	O
Right Atrium	Right_Atrium	HEART	PrimaryTissue	O
Right Ventricle	Right_Ventricle	HEART	PrimaryTissue	O
iPS-20b Cell Line	iPS-20b_Cell_Line	IPSC	CellLine	O
iPS DF 6.9 Cell Line	iPS_DF_6.9_Cell_Line	IPSC	CellLine	O
iPS DF 19.11 Cell Line	iPS_DF_19.11_Cell_Line	IPSC	CellLine	O
iPS-15b Cell Line	iPS-15b_Cell_Line	IPSC	CellLine	O
iPS-18 Cell Line	iPS-18_Cell_Line	IPSC	CellLine	O
Fetal Kidney	Fetal_Kidney	KIDNEY	PrimaryTissue	O
Liver	Adult_Liver	LIVER	PrimaryTissue	O
Psoas Muscle	Psoas_Muscle	MUSCLE	PrimaryTissue	O
Fetal Muscle Trunk	Fetal_Muscle_Trunk	MUSCLE	PrimaryTissue	O
HSMM Skeletal Muscle Myoblasts Cell Line	HSMM_Skeletal_Muscle_Myoblasts	MUSCLE	CellLine	O
Muscle Satellite Cultured Cells	Muscle_Satellite_Cultured_Cells	MUSCLE	PrimaryCell	O
Skeletal Muscle Female	Skeletal_Muscle_Female	MUSCLE	PrimaryTissue	O
Skeletal Muscle Male	Skeletal_Muscle_Male	MUSCLE	PrimaryTissue	O
Fetal Muscle Leg	Fetal_Muscle_Leg	MUSCLE_LEG	PrimaryTissue	O
Ovary	Ovary	OVARY	PrimaryTissue	O
Pancreas	Pancreas	PANCREAS	PrimaryTissue	O
Pancreatic Islets	Pancreatic_Islets	PANCREAS	PrimaryTissue	O
Placenta	Fetal_Placenta	PLACENTA	PrimaryTissue	O
Placenta Amnion	Placenta_Amnion	PLACENTA	PrimaryTissue	O
NHDF-Ad Adult Dermal Fibroblast Primary Cells	NHDF-Ad_Adult_Dermal_Fibroblasts	SKIN	PrimaryCell	O
NHEK-Epidermal Keratinocyte Primary Cells	NHEK-Epidermal_Keratinocytes	SKIN	PrimaryCell	O
Foreskin Fibroblast Primary Cells skin01	Penis_Foreskin_Fibroblast_Primary_Cells_skin01	SKIN	PrimaryCell	O
Foreskin Fibroblast Primary Cells skin02	Penis_Foreskin_Fibroblast_Primary_Cells_skin02	SKIN	PrimaryCell	O
Foreskin Keratinocyte Primary Cells skin02	Penis_Foreskin_Keratinocyte_Primary_Cells_skin02	SKIN	PrimaryCell	O
Foreskin Keratinocyte Primary Cells skin03	Penis_Foreskin_Keratinocyte_Primary_Cells_skin03	SKIN	PrimaryCell	O

Foreskin Melanocyte Primary Cells skin01	Penis_Foreskin_Melanocyte_Primary_Cells_skin01	SKIN	PrimaryCell	O
Foreskin Melanocyte Primary Cells skin03	Penis_Foreskin_Melanocyte_Primary_Cells_skin03	SKIN	PrimaryCell	O
Spleen	Spleen	SPLEEN	PrimaryTissue	O
Bone Marrow Derived Cultured Mesenchymal Stem Cells	Bone_Marrow_Derived_Mesenchymal_Stem_Cell_Cultured_Cells	STROMAL_CONNECTIVE	PrimaryCell	O
Mesenchymal Stem Cell Derived Chondrocyte Cultured Cells	Chondrocytes_from_Bone_Marrow_Derived_Mesenchymal_Stem_Cell_Cultured_Cells	STROMAL_CONNECTIVE	PrimaryCell	O
Thymus	Thymus	THYMUS	PrimaryTissue	O
Aorta	Aorta	VASCULAR	PrimaryTissue	O
HUVEC Umbilical Vein Endothelial Primary Cells	HUVEC_Umbilical_Vein_Endothelial_Cells	VASCULAR	PrimaryCell	O
Primary Natural Killer cells from peripheral blood	CD56_Primary_Cells	BLOOD	PrimaryCell	T
Primary T CD8+ memory cells from peripheral blood	CD8_Memory_Primary_Cells	BLOOD	PrimaryCell	T
Primary T CD8+ naive cells from peripheral blood	CD8_Naive_Primary_Cells	BLOOD	PrimaryCell	T
Primary T cells effector/memory enriched from peripheral blood	CD4+_CD25int_CD127+_Tmem_Primary_Cells	BLOOD	PrimaryCell	T
Primary T cells from cord blood	CD3_Primary_Cells_Cord_BI	BLOOD	PrimaryCell	T
Primary T cells from peripheral blood	CD3_Primary_Cells_Peripheral_UW	BLOOD	PrimaryCell	T
Primary T helper cells from peripheral blood	CD4+_CD25-_Th_Primary_Cells	BLOOD	PrimaryCell	T
Primary T helper cells PMA-I stimulated	CD4+_CD25-_IL17-_PMA-Ionomycin_stimulated_MACS_purified_Th_Primary_Cells	BLOOD	PrimaryCell	T
Primary T helper memory cells from peripheral blood 1	CD4+_CD25-_CD45RO+_Memory_Primary_Cells	BLOOD	PrimaryCell	T
Primary T helper memory cells from peripheral blood 2	CD4_Memory_Primary_Cells	BLOOD	PrimaryCell	T
Primary T helper naive cells from peripheral blood	CD4_Naive_Primary_Cells	BLOOD	PrimaryCell	T
Primary T helper naive cells from peripheral blood	CD4+_CD25-_CD45RA+_Naive_Primary_Cells	BLOOD	PrimaryCell	T
Primary T helper 17 cells PMA-I stimulated	CD4+_CD25-_IL17+_PMA-Ionomcyin_stimulated_Th17_Primary_Cells	BLOOD	PrimaryCell	Th17
Primary T regulatory cells from peripheral blood	CD4+_CD25+_CD127-_Treg_Primary_Cells	BLOOD	PrimaryCell	Treg

Key

Standardized Epigenome name	Name of cell line
Epigenome name (from EDACC Release 9 directory)	alias
ANATOMY	cell type or tissue of procedence
TYPE	primary or established cell line
Cell Bucket for Aanalysis	Major cell type associated to cell line for this analysis
B:	B cells
T:	T cells
M:	Monocytes/macrophages
O:	Other
L:	Lung

Supplementary Table 6. Assignment of regulatory features into broad categories for this analysis

Histone modification or variant	Putative functions
H2az	Histone protein variant (H2A.Z) associated with regulatory elements with dynamic chromatin
H3k4me1	Mark of regulatory elements associated with enhancers and other distal elements, but also enriched downstream of transcription starts
H3k04me1	Mark of regulatory elements associated with enhancers and other distal elements, but also enriched downstream of transcription starts
H3k4me2	Mark of regulatory elements associated with promoters and enhancers
H3k04me2	Mark of regulatory elements associated with promoters and enhancers
H3k4me2	Mark of regulatory elements associated with promoters and enhancers
H3k04me2	Mark of regulatory elements associated with promoters and enhancers
H3k4me3	Mark of regulatory elements primarily associated with promoters/transcription starts
H3k04me3	Mark of regulatory elements primarily associated with promoters/transcription starts
H3k04me3Ohtam	Mark of regulatory elements primarily associated with promoters/transcription starts
H3k9ac	Mark of active regulatory elements with preference for promoters
H3k09ac	Mark of active regulatory elements with preference for promoters
H3k9me1	Preference for the 5’ end of genes
H3k09me1	Preference for the 5’ end of genes
H3k9me3	Repressive mark associated with constitutive heterochromatin and repetitive elements
H3k09me3	Repressive mark associated with constitutive heterochromatin and repetitive elements
H3k27ac	Mark of active regulatory elements; may distinguish active enhancers and promoters from their inactive counterparts
H3k27ac	Mark of active regulatory elements; may distinguish active enhancers and promoters from their inactive counterparts
H3k27me3	Repressive mark established by polycomb complex activity associated with repressive domains and silent developmental genes
H3k36me3	Elongation mark associated with transcribed portions of genes, with preference for 3’ regions after intron 1
H3k79me2	Transcription-associated mark, with preference for 5’ end of genes
H4k20me1	Preference for 5’ end of genes
Active TSS	
Flanking Active TSS	
Transcr. at gene 5' and 3'	Transcription/Elongation marker observed
Strong transcription	Transcription/Elongation marker observed
Weak transcription	Transcription/Elongation marker observed
Genic enhancers	
Enhancers	
ZNF genes & repeats	ZNF genes harbor 3' repeats and have promoter and elongation markers. However, they don't seem to promote any gene activity tested.
Heterochromatin	Dead zone
Bivalent/Poised TSS	Inactive Promoter
Flanking Bivalent TSS/Enh	Inactive Enhancer
Bivalent Enhancer	Inactive Enhancer
Repressed PolyComb	
Weak Repressed PolyComb	
Quiescent/Low	No/low marker activity

**Key**	
Histone modification or variant	Type of regulatory element or histone modification
Putative functions	predicted regulatory function
Bucket_for_analysis	Major regulatory function associated to element for this analysis
