# Supplementary material for: A systems biology approach uncovers cell-specific gene regulatory effects of genetic associations in multiple sclerosis
Source: Nat Commun. 2019 May 20;10:2236. doi: 10.1038/s41467-019-09773-y (PMC6527683; doi:10.1038/s41467-019-09773-y)
Supplement: Supplementary file 3 — Description of Additional Supplementary Files [file 41467_2019_9773_MOESM3_ESM.pdf]

Description of Additional Supplementary Files

Supplementary Dataset 1: Master Table containing regulatory features for each SNP in each cell type used in this work.

Supplementary Dataset 2. Predicted regulatory effects of genes (GW_Bcells_r^2>0.5)

Supplementary Dataset 3. Predicted regulatory effects of genes (GW_ CNS_r^2>0.5)

Supplementary Dataset 4. Predicted regulatory effects of genes (GW_ Lung_r^2>0.5)

Supplementary Dataset 5. Predicted regulatory effects of genes (GW_ Monocytes_r^2>0.5)

Supplementary Dataset 6. Predicted regulatory effects of genes (GW_ T cells_r^2>0.5)

Supplementary Dataset 7. Predicted regulatory effects of genes (GW_ Th1 cells_r^2>0.5)

Supplementary Dataset 8. Predicted regulatory effects of genes (GW_ Th2 cells_r^2>0.5)

Supplementary Dataset 9. Predicted regulatory effects of genes (GW_ Th17 cells_r^2>0.5)

Supplementary Dataset 10. Predicted regulatory effects of genes (GW_ Treg cells_r^2>0.5)

Supplementary Dataset 11. Predicted regulatory effects of genes (SR_ Bcells_r^2>0.5)

Supplementary Dataset 12. Predicted regulatory effects of genes (SR_ CNS_r^2>0.5)

Supplementary Dataset 13. Predicted regulatory effects of genes (SR_ Lung_r^2>0.5)

Supplementary Dataset 14. Predicted regulatory effects of genes (SR_ Monocytes_r^2>0.5)

Supplementary Dataset 15. Predicted regulatory effects of genes (SR_ T cells_r^2>0.5)

Supplementary Dataset 16. Predicted regulatory effects of genes (SR_ Th1 cells_r^2>0.5)

Supplementary Dataset 17. Predicted regulatory effects of genes (SR_ Th2 cells_r^2>0.5)

Supplementary Dataset 18. Predicted regulatory effects of genes (SR_ Th17 cells_r^2>0.5)

Supplementary Dataset 19. Predicted regulatory effects of genes (SR_ Treg cells_r^2>0.5)

Supplementary Dataset 20. Predicted regulatory effects of genes (NR_B cells_r^2>0.5)

Supplementary Dataset 21. Predicted regulatory effects of genes (NR_CNS_r^2>0.5)

Supplementary Dataset 22. Predicted regulatory effects of genes (NR_Lung_r^2>0.5)

Supplementary Dataset 23. Predicted regulatory effects of genes (NR_Monocytes_r^2>0.5)

Supplementary Dataset 24. Predicted regulatory effects of genes (NR_T cells_r^2>0.5)

Supplementary Dataset 25. Predicted regulatory effects of genes (NR_Th1 cells_r^2>0.5)

Supplementary Dataset 26. Predicted regulatory effects of genes (NR_Th2 cells_r^2>0.5)

Supplementary Dataset 27. Predicted regulatory effects of genes (NR_Th17 cells_r^2>0.5)

Supplementary Dataset 28. Predicted regulatory effects of genes (NR_Treg cells_r^2>0.5)

Supplementary Dataset 29. Prioritized genes by region and cell/tissue type

Supplementary Dataset 30: Network properties of MS associated genes (population level)

Supplementary Dataset 31: Analysis of MS associated SNPs in a sub-set of 2,370 patients and 412 controls from UCSF

Supplementary Dataset 32. Network properties of MS associated genes (individual level)

Supplementary Dataset 33. Autosomal non-MHC genome-wide effects.

Supplementary Dataset 34. Strongly suggestive list of autosomal non-MHC loci.

Supplementary Dataset 35. List of non-replicated autosomal non-MHC effects.
